# RNAseq analysis of fast skeletal muscle in restriction-fed transgenic coho salmon (*Oncorhynchus kisutch*): an experimental model uncoupling the growth hormone and nutritional signals regulating growth

**DOI:** 10.1186/s12864-015-1782-z

**Published:** 2015-07-31

**Authors:** Daniel Garcia de la serrana, Robert H Devlin, Ian A Johnston

**Affiliations:** Scottish Oceans Institute, School of Biology, University of St Andrews, KY16 8LB St Andrews, Scotland UK; Department of Fisheries and Oceans, Centre for Aquaculture and Environmental Research, 4160 Marine Drive, West Vancouver, BC V7V 1N6 Canada

**Keywords:** Muscle growth, teleost fish, Growth hormone transgenics, Skeletal muscle transcriptome, Fish nutrition

## Abstract

**Background:**

Coho salmon (*Oncorhynchus kisutch*) transgenic for growth hormone (Gh) express Gh in multiple tissues which results in increased appetite and continuous high growth with satiation feeding. Restricting Gh-transgenics to the same lower ration (TR) as wild-type fish (WT) results in similar growth, but with the recruitment of fewer, larger diameter, muscle skeletal fibres to reach a given body size. In order to better understand the genetic mechanisms behind these different patterns of muscle growth and to investigate how the decoupling of Gh and nutritional signals affects gene regulation we used RNA-seq to compare the fast skeletal muscle transcriptome in TR and WT coho salmon.

**Results:**

Illumina sequencing of individually barcoded libraries from 6 WT and 6 TR coho salmon yielded 704,550,985 paired end reads which were used to construct 323,115 contigs containing 19,093 unique genes of which >10,000 contained >90 % of the coding sequence. Transcripts coding for 31 genes required for myoblast fusion were identified with 22 significantly downregulated in TR relative to WT fish, including 10 (*vaspa*, *cdh15*, *graf1*, *crk*, *crkl*, *dock1*, *trio*, *plekho1a*, *cdc42a* and *dock5*) associated with signaling through the cell surface protein cadherin. Nineteen out of 44 (43 %) translation initiation factors and 14 of 47 (30 %) protein chaperones were upregulated in TR relative to WT fish.

**Conclusions:**

TR coho salmon showed increased growth hormone transcripts and gene expression associated with protein synthesis and folding than WT fish even though net rates of protein accretion were similar. The uncoupling of Gh and amino acid signals likely results in additional costs of transcription associated with protein turnover in TR fish. The predicted reduction in the ionic costs of homeostasis in TR fish associated with increased fibre size were shown to involve multiple pathways regulating myotube fusion, particularly cadherin signaling.

**Electronic supplementary material:**

The online version of this article (doi:10.1186/s12864-015-1782-z) contains supplementary material, which is available to authorized users.

## Background

The growth hormone (Gh) axis in coho salmon (*Oncorhynchus kisutch*) and other teleosts is normally tightly coupled to energy intake and is modulated by a large number of environmental factors [1]. The normal feedback control systems are essentially disabled in growth hormone-transgenics due to the extra-pituitary expression of Gh in other tissues [2, 3]. High constitutive expression of *Gh* results in increased aggressiveness, appetite and foraging activity [4, 5] leading to continuous fast growth when fish are fed to satiation [6]. Gh stimulates insulin-like growth factor-1 (Igf1) secretion in the liver. Circulating Igfs bind to Igf receptors and activate the signalling cascades that regulate protein synthesis. Plasma Igf1 and amino acids from the diet further stimulate Igf synthesis in muscle and other tissues via paracrine action [7, 8]. Gh-transgenics exhibit increased plasma insulin-like growth factor-1 (Igf1; ~25 ng/ml), tissue *igf1* mRNA and *Gh-receptor* (*ghr*) levels relative to wild-type fish (circulating Igf1 was ~8 ng/ml in WT) [3]. Feeding coho salmon Gh-transgenics below appetite (restriction-fed transgenics, TR) with a similar ration size as wild-type fish (WT) results in an uncoupling of the growth hormone and nutritional signals regulating growth. TR fish grow more slowly and show lower circulating Igf1 than Gh-transgenic fish fed to satiation (TF) (10 ng/ml in TR compared with 25 ng/ml to TF animals), which indicates the nutritional regulation of *igf1* expression remains functional at the target tissue level [3]. TR animals showed higher circulating Gh (8 ng/ml) levels and unchanged *ghr* mRNA relative to Gh-transgenics fed to satiation (TF) (4 ng/ml circulating Gh), but retained higher feeding motivation and foraging activity than WT [3].

Fast skeletal muscle fibres are formed in teleost fish immediately following somite formation and then in the juvenile/adult stages by processes of stratified and mosaic hyperplasia (MH) [9, 10]. Myoblast-myoblast fusion results in the formation of a nascent myotube which elongates and gains additional nuclei through myoblast-myotube fusion. Activation of terminal differentiation and sarcomere formation then results in the production of an immature muscle fibre. Typically, myotube production in fast muscle continues until the fish reaches around 40-50 % of their maximum attainable adult body length [11].

Hill et al. [12] provided indirect evidence that Gh-transgenic coho salmon fed to satiation showed enhanced muscle growth by hyperplasia. In a recent study we tested this hypothesis by measuring the number and size distribution of fast myotomal muscle fibres in three groups of coho salmon of similar body length: 1-year old Gh-transgenics fed to satiation (TF), 2-year old restriction-fed Gh-transgenics (TR) and wild type (WT) [13]. TF coho salmon recruited fast muscle fibres at twice the rate as WT fish, but showed a similar contribution of hyperplasia and hypertrophy to reach a given body length i.e. the hypothesis of an increased importance of hyperplasia in transgenics was not supported. Unexpectedly, TR recruited 49 % fewer fibres with a 20 % higher fibre diameter than either WT or TF fish and had larger diameter fibres across the whole range of fibre sizes, i.e. increased hypertrophy was evident for all cohorts of fibres produced during ontogeny [13]. There is direct experimental evidence from inhibitor and Nuclear Magnetic Resonance (NMR) studies that larger diameter muscle fibres have lower costs of ionic homeostasis than smaller ones due to their lower surface to volume (S/V) ratio [14, 15]. Thus the ~40 % reduction in fibre S/V ratio in TR relative to WT fish would be expected to produce proportional savings in routine maintenance costs [13]. Previously we suggested that adjustments in fibre size might permit the reallocation of energy from maintenance to locomotion which would help explain why calorie-restricted transgenics grow at the same rate as WT fish whilst exhibiting markedly higher activity levels [13].

Preliminary, studies indicated that several genes associated with myotube formation were downregulated in TR relative to WT fish [13]. In order to gain a broader understanding of fast skeletal muscle gene regulation in TR compare to WT fish we have now carried out RNA-seq in 6 TR and 6 WT individual coho salmon for which we had associated data on the number and size of fast muscle fibres [13], and validated the results by qPCR analysis. RNA was sequenced using Illumina HiSeq2000 and mapped reads (DESEQ normalized counts) were used to study global differences in digital gene expression (DGE) between groups. Following enriched Gene Ontology (GO) analysis we tested specific hypotheses about the effects of uncoupling growth hormone and nutrient signals by analysing the expression of genes related to growth hormone signalling, protein translation, protein-folding and myoblast fusion.

## Results and discussion

### Transcriptome analysis

Total RNA from TR and WT fish (*n* = 6 per condition) with known differences in muscle cellularity [13] were sequenced in 2 lines of Illumina Hiseq2000 . A total of 704,550,985 paired-end reads were *de novo* assembled into 323,115 contigs with average length >1,000 bp and N_50_ > 2,000 bp (Table 1; Additional file 1). Using BLASTx against the NCBI non-redundant (nr) database we successfully identified a total of 153,839 contigs and, from them, a total of 149,738 were also annotated with Gene Ontology (GO) terms (Table 1). A total of 19,093 unique genes were identified in the fully annotated transcriptome (Additional file 1). The teleost linage underwent a whole-genome duplication (WGD) relative to the last common ancestor with humans around 350-320Mya [16], increasing gene content by 15-20 % [17]. The salmonids experienced a fourth lineage-specific WGD (4R) around 88Mya, and approximately 50 % of the resulting paralogues are still present in extant species [18]. Several examples of gene expansion due to the salmonid WGD have been described in detail [19, 20]. In order to estimate the representation of 4R paralogues in the transcriptome 38 well-characterized Atlantic salmon (*Salmo salar*) paralogues gene families were blasted (tBLASTn) against the coho salmon de novo transcriptome. Seventeen out of 38 paralogues were unequivocally identified (44 %) and three more appeared with very low e-values (Table 2), though not all can be expected to be expressed in skeletal muscle. 21,995 protein-coding genes have been reported for the human genome of which 12,557 are expressed in the muscle transcriptome [21]. The 51 % higher gene content of the coho skeletal muscle transcriptome (19,093) than in humans likely reflects this history of the succesive WGD. Furthermore, the continuous expression of Gh might also increase the number of genes expressed at detectable levels in the muscle.

**Table 1:** Sequencing and *de novo* Trinity assembly metrics

	WT	TR
Paired-end reads	*351,369,759*	*353,181,226*
Reads assembly	*267,224,210*	*254,344,286*
Percentage assembly	*71.53*	*72.04*
Contigs	*323,115*	
Average length (bp)	*1,103*	
N50 (bp)	*2,142*	
Components annotated	*149,738*	
Unigenes	*19,093*	
Complete CDS	*10,030*	
KAAS annotated maps	*309*	

TR: restriction-fed growth hormone-transgenic coho salmon; WT: wild-type coho salmon; N50: average length that contains at least half of the sum of the lengths for all contigs; bp: base pair; CDS: coding sequence; KAAS: KEEG automatic annotation service

**Table 2:** Estimation of paralogues content in the coho salmon transcriptome

*S. salar* paralogue	Coho salmon contig	E-value	Id (%)	Alignment (AA)
*igfbp1a1*	comp120142_c0_seq3	0	95	807
*igfbp1a2*	n/a	n/a	n/a	n/a
*igfbp1b1*	n/a	n/a	n/a	n/a
*igfbp1b2*	n/a	n/a	n/a	n/a
*igfbp2a*	n/a	n/a	n/a	n/a
*igfbp2b1*	n/a	n/a	n/a	n/a
*igfbp2b2*	comp132077_c2_seq30	0.24	100	20
*igfbp3a1*	comp125786_c0_seq1	0	96	889
*igfbp3a2*	n/a	n/a	n/a	n/a
*igfbp3b1*	n/a	n/a	n/a	n/a
*igfbp3b2*	comp712690_c0_seq1	1.00E-154	96	324
*igfbp4*	n/a	n/a	n/a	n/a
*igfbp5a*	n/a	n/a	n/a	n/a
*igfbp5b1*	comp126977_c0_seq9	0	96	807
*igfbp5b2*	n/a	n/a	n/a	n/a
*igfbp6a1*	n/a	n/a	n/a	n/a
*igfbp6a2*	n/a	n/a	n/a	n/a
*igfbp6b1*	n/a	n/a	n/a	n/a
*igfbp 6b2*	comp130388_c0_seq9	0	98	609
*akirin 1(1a)*	comp126876_c0_seq6	0	96	638
*akirin 1(1b)*	comp126876_c0_seq2	0	96	655
*akirin 1(2a)*	comp128245_c1_seq9	0	95	690
*akirin 1(2b)*	comp128245_c1_seq10	0	98	592
*akirin 2(1a)*	n/a	n/a	n/a	n/a
*akirin 2(1b)*	comp127034_c0_seq3	0	98	472
*akirin 2(2a)*	n/a	n/a	n/a	n/a
*akirin 2(2b)*	comp126525_c1_seq7	0	98	546
*hsp90aa1.3*	comp132154_c0_seq29	0	92	1386
*hsp90aa1.4*	comp132154_c0_seq71	0	95	1281
*hsp90aa1.1*	n/a	n/a	n/a	n/a
*hsp90aa1.2*	n/a	n/a	n/a	n/a
*hsp90b1*	comp122350_c0_seq5	0	97	619
*hsp90b2*	n/a	n/a	n/a	n/a
*aqp8*	comp132382_c1_seq19	1.00E-28	95	82
*aqp8aa1*	comp104094_c0_seq1	0	91	796
*aqp8ab*	n/a	n/a	n/a	n/a
*aqp8b*	comp450295_c0_seq1	0.87	100	19
*myod1*	comp122414_c0_seq4	0	96	986
*myod2*	n/a	n/a	n/a	n/a
*myo1c*	comp131066_c1_seq17	0	98	859

Id: percentage of identity at amino acid level between *S.salar* and *O.kitsuch* orthologues

Alignment: Total number of amino acids successfully aligned during the tBLASTn between *S.salar* and *O.kitsuch* orthologues

n/a: Paralogues not identified in the transcriptome

Annotated contigs were blasted against the KEGG database using the online web-server KAAS to map them against the main metabolic and signaling pathways, yielding a total of 309 KEGG maps where coho salmon contigs were present (Table 1; Hierarchical file with all the details of the KEGG mapped contigs can be found in Additional file 1). Comparative analysis between annotated contigs against the annotated zebrafish proteome (*Danio rerio*) allowed us to identify 10,030 genes estimated to contain over 90 % of the predicted coding sequence (CDS) (Additional file 2). We also found that 1000 contigs were over >100 % of the CDS, indicating that some coho salmon genes were between 1-20 % longer than their zebrafish orthologues (Additional file 2).

### Digital Gene Expression (DGE)

Paired-end reads from individual fish libraries were mapped against the *de novo* transcriptome (Additional file 3). Mapped reads were normalized by contig length and library depth following a negative binomial using DESEQ and used for DGE analysis. Global DGE comparison between TR and WT yielded an initial list of 384 contigs that were differentially expressed (FDR < 0.05). Contigs with <15 normalized reads mapped and a fold-change <2log_2_ were discarded. Redundant contigs for all genes from the initial global DGE list were identified in the annotated transcriptome and their DESEQ-counts values were individually investigated. Those genes for which all-redundant “sister” contigs were found to show consistent, but not necessarily significant, changes in expression between groups were retained (see Methods). Care was taken to identify 4R and 3R-paralogues that might be annotated with the same ID by exploring the alignment of the sister contigs. After curation for redundancy and quality control, a total of 186 genes from TR and 199 from WT were considered to be differentially expressed (Additional file 4).

RNAseq has many advantages, but it does present challenges with respect to statistical analysis and interpretation. Firstly, transcription is not tightly coupled to translation in eukaryotes, complicating inferences about the functional significance of changes in transcript abundance. Secondly, the activity of signalling pathways is often dependent on posttranslational modifications such as phosphorylation and/or changes in compartmentalisation within the cell. In addition, functional interpretations based on transcript abundance cannot distinguish between mRNA transcription vs degradation rates. It is also easy to fall into the trap of providing a series of *post hoc* stories when reporting transcriptomic data rather than testing *a priori* hypotheses. Standard corrections for multiple testing coupled with long gene lists also leads to a very high barrier for establishing statistical significance, leading to type-2 statistical errors.

**Fig. 1:**
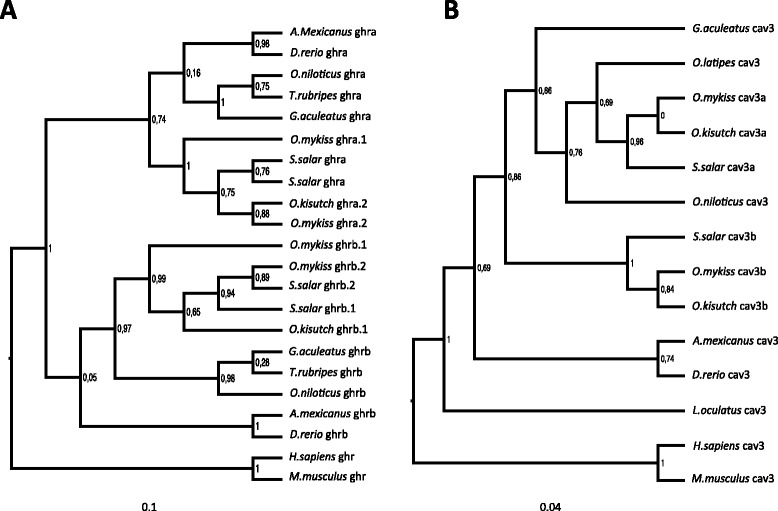
Maximum likelihood phylogenetic trees for two 4R paralogues. **a** . *Growth hormone receptor* (*ghr*) **b** . *Caveolin 3* (*cav3*). Posterior values are indicated in the nodes of each branch. Species abbreviations are as follow; *S. salar*: *Salmo salar*; *O. mykiss*: *Oncorhynchus mykiss*; *O. kisutch*: *Oncorhynchus kisutch*; *A. mexicanus*: *Astyanax mexicanus*; *D. rerio*: *Danio rerio*; *T.rubripes*: *Takifugu rubripes*; *G. aculeatus*: *Gasterosteus aculeatus*; *O. Niloticus*: *Oreochromis niloticus*; *H. sapiens*: *Homo sapiens*; *M. musculus*: *Mus musculus*

We therefore used a multistep process to analyse the DGE results from TR and WT fish fed the same ration. Firstly, enriched Gene Ontology (GO) analysis was used to identify initial categories of those genes obtained from the global analysis for further investigation (Additional file 4). For the TR fish, GO analysis revealed an enrichment in genes related to “macromolecule metabolic process” (GO:44260), “RNA metabolic process” (GO:16070), “regulation of translation” (GO:6417), “translation factor activity, nucleic acid binding” (GO:8135) or “RNA binding” (GO:3723) (FDR < 0.05; Table 3). WT animals had a higher expression of genes related to “autophagy” (GO:6914), “cellular metabolic process” (GO:44237), “catabolic process” (GO:9056) and “vacuole organization” (GO:7033) (FDR < 0.05; Table 3). Based on GO enrichment analysis and previous works [13] we decided to specifically test the expression of genes related to myoblast fusion, protein synthesis, protein degradation by the proteasome system, protein folding and also the Gh-Igf receptors and ligands. KAAS annotation results and a literature survey of the results of functional analyses were used to identify the genes to include. 363 genes were identified in total (Additional file 4), including 4R and 3R paralogues where identified (examples of 4R salmonid paralogues are in Fig. 1; trees for the potential 4R paralogues identified can be found in Additional file 5). DESEQ-counts (considered more stringent and a better estimation of gene expression than FPKM) from individual animals for each gene were then retrieved in order to test specific *a priori* hypotheses.

**Table 3:** Gene ontology analysis

Condition	GO term	GO:ID	Number of genes	FDR
TR				
*Biological process*	Ribonucleoprotein complex biogenesis	0022613	18	7.1e^-8^
	RNA processing	0006396	21	6.0e^-5^
	Regulation of translational initiation	0006446	8	1.7e^-4^
	Cellular macromolecule metabolic process	0044260	70	2.0e^-4^
	Cellular nitrogen compounds metabolic process	0034641	64	3.7e^-4^
	Primary metabolic process	0044238	84	3.7e^-4^
*Molecular function*	RNA binding	0003723	54	4.3e^-20^
	Nucleic acid binding	0003676	58	1.4e^-6^
	Protein binding	0005515	89	2.9e^-3^
	Organic cyclic compound binding	0097159	64	7.4e^-3^
	Catalytic activity	0003824	49	0.05
*Cellular component*	Ribonucleoprotein complex	0030529	22	3.8e^-7^
	Nuclei	0005634	82	1.9e^-6^
	Nucleolus	0005730	22	2.5e^-5^
	Intracellular organelle	0044446	108	2.5e^-5^
WT				
*Biological process*	Macroautophagy	0016236	7	1.5e^-4^
	Cellular protein modification process	0006464	37	5.5e^-4^
	Autophagy	0006914	9	5.5e^-4^
	Macromolecule modification	0043412	36	3.4e^-3^
	Cellular response to nutrient levels	0031669	8	4.5e^-3^
	Protein modification by small protein conjugation	0032446	16	4.8e^-3^
*Molecular function*	Protein binding	0005515	97	1.1e^-3^
	Catalytic activity	0003824	54	2.8e^-2^
*Cellular component*	Vacuole	0005773	21	1.0e^-6^
	Cytoplasm	0005737	112	1.0e^-6^
	Lysosome	0005764	17	5.0e^-5^
	Intracellular component	0044424	121	6.1e^-5^

Gene Ontology analysis from genes with significant differences in expression between restriction-fed growth hormone-transgenic coho salmon (TR) and wild-type coho salmon (WT); GO: Gene Ontology; FDR: False discovery rate

**Fig. 2:**
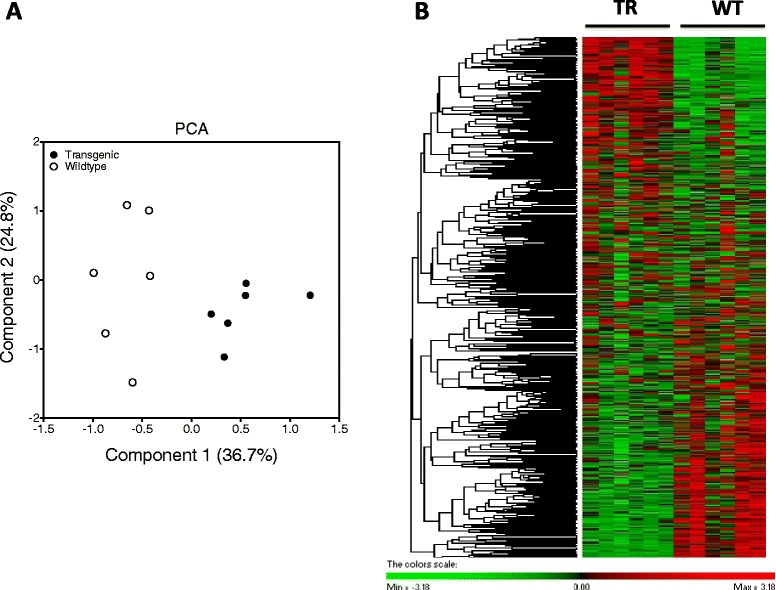
Principal components analysis (PCA) and hierarchical heat map cluster of all the genes manually analysed for DGE. **a** . Principal Components Analysis of the DGE from all 363 genes analysed to test our specific hypothesis. In brackets is indicated the percentage of variation explained by each of the components. Circles represent individual fish from restricted diet transgenics (full circles) and wild-type (empty circles) groups **b** . Hierarchical heat map cluster showing individual DGE values for TR (restricted diet transgenic) and WT (wild-type) coho salmon. Rows are standardized to have a mean of 0 and standard deviation of 1; red indicates high and green indicates low DGE values. Each rectangle represents individual DGE values

Before analysing in detail the differences in DGE between genes a Principal Components Analysis (PCA) was carried to determine if the categories selected were informative of the differences between TR and WT individuals (Fig. 2a). Components 1 and 2 generated from PCA analysis explained 61.5 % of the variability of the data and were able to distinguish TR and WT in two separated clusters (Fig. 2a). This is an indication that the data used was informative with respect to the differences between groups, which is highly apparent in the hierarchical clustering shown in Fig. 2b.

### Growth hormone system and protein synthesis

**Fig. 3:**
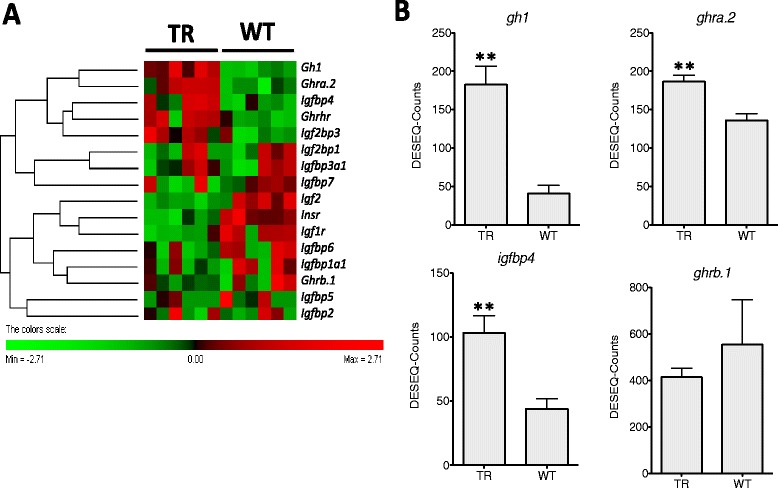
Gh and Igf receptors and ligands. **a** . Hierarchical heat map cluster showing individual DGE values for TR (restricted diet transgenic) and WT (wild-type) coho salmons. Rows are standardized to have a mean of 0 and standard deviation of 1; red indicates high and green indicates low DGE values. Each rectangle represents individual DGE values **b** . Bar graphs showing DGE values from TR and WT groups (mean ± SE) for *growth hormone* (*gh1*), *growth hormone receptor a.2* (*ghra.2*), *insulin-like growth factor binding protein 4* (*igfbp4*) and *growth hormone receptor b.1 (ghrb.1;* in this particular case differences between TR and WT are not significant and the gene is show in order to compare with *ghra.2*). Significant differences are indicated with one (FDR < 0.05) or two asterisk (FDR < 0.01) according to the degree of significance

Nineteen genes associated with the growth hormone system (*gh*, *igf*, their receptors and binding proteins) were identified in the transcriptome and filtered for redundancy as previously described. In order to capture all the biological relevant changes, the requirement that differences should be higher than 2log_2_-fold change was relaxed but not the requirement that average DESEQ-counts should be >15. As expected due to the transgenic nature of the coho salmon, *gh1* gene transcripts were 4.5-fold higher in TR compared to WT [3] (Fig. 3). Similarly, previous studies have also reported an increase in *growth hormone receptor* (*ghr*) expression in muscle [3]. However, that study didn’t distinguish between ghr paralogues whereas we identified *ghra.2* and *ghrb.1*, two of the potential 4 salmonid paralogues for the receptor (Figs. 1 and 3; Additional file 6). We found that *ghra.2* was 1.4-fold higher in TR than WT fish (Fig. 3b) whereas *ghrb.1* showed unaltered expression, indicating differential regulation of paralogues. *Igf2*/*igf1r* and the insulin receptor (*inr*) were downregulated 2- and 3-fold respectively (FDR < 0.01) in TR than WT fish, most likely as part of a negative feedback response to increased growth hormone expression (Fig. 3; Additional file 6).

**Fig. 4:**
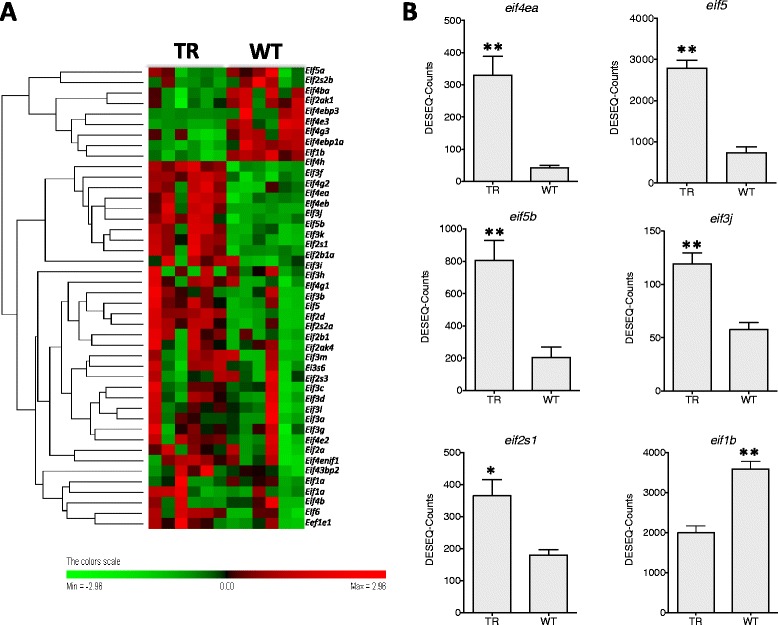
Elongation factors and eukaryotic translation factors DGE. **a** . Hierarchical heat map cluster showing individual DGE values for TR (restricted diet transgenic) and WT (wild-type) coho salmon. Rows are standardized to have a mean of 0 and standard deviation of 1; red indicates high and green indicates low DGE values. Each rectangle represents individual DGE values **b** . Bar graphs showing DGE values from TR and WT groups (mean ± SE) for *eukaryotic translation initiation factor 4E* (*eif4e*), *eukaryotic translation initiation factor 5* (*eif5*), *eukaryotic translation initiation factor 5b* (*eif5b*), *eukaryotic translation initiation factor 3j* (*eif3j*), *eukaryotic translation initiation factor 2 s1* (*eif2s1*) and *eukaryotic translation initiation factor 1b* (*eif1b*). Significant differences are indicated with one (FDR < 0.05) or two asterisk (FDR < 0.01) according to the degree of significance

**Fig. 5:**
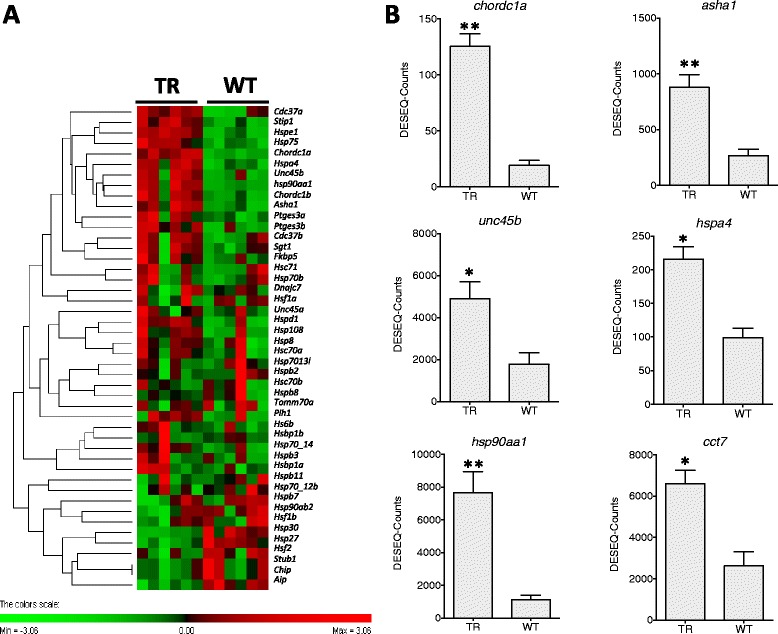
Chaperones, co-chaperones and stress factors DGE. **a** . Hierarchical heat map cluster showing individual DGE values for TR (restricted diet transgenic) and WT (wild-type) coho salmon. Rows are standardized to have a mean of 0 and standard deviation of 1; red indicates high and green indicates low DGE values. Each rectangle represents individual DGE values **b** . Bar graphs showing DGE values from TR and WT groups (mean ± SE) for *Unc-45B homologue* (*unc45b*), *cysteine and histidine-rich domain containing 1* (*chordc1*), *activator of heat shock 90 kDa protein ATPase homolog 1* (*asha1*), *heat shock protein 4* (*hspa4*), *cytosolic heat shock protein 90 alpha* (*hsp90aa1*), *chaperonin containing TCP1 and subunit 7* (*cct7*). Significant differences are indicated with one (FDR < 0.05) or two asterisk (FDR < 0.01) according to the degree of significance

**Fig. 6:**
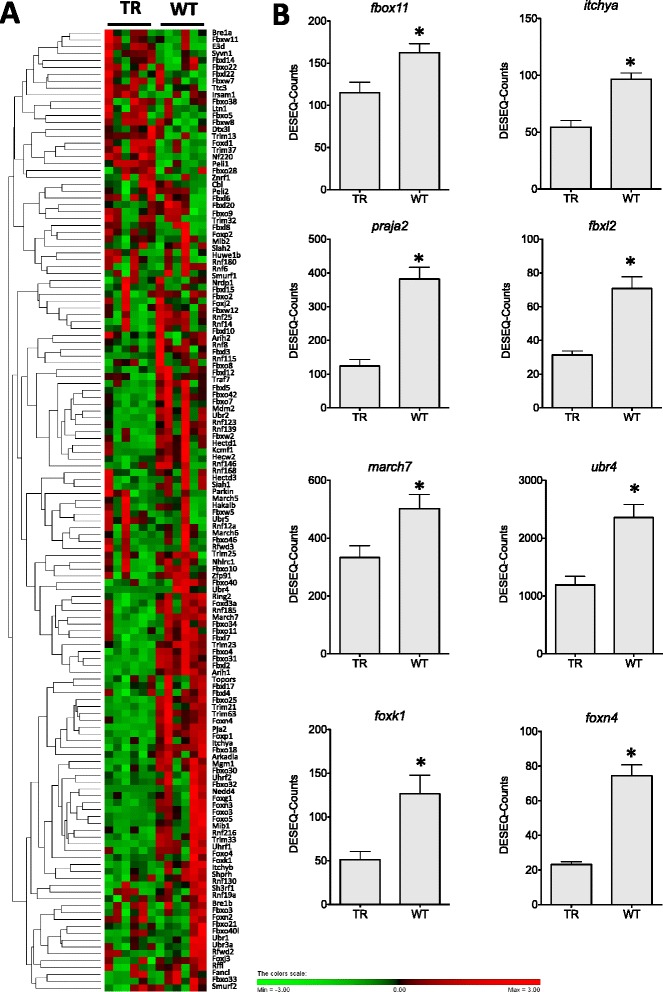
E3-ubiquitin ligases DGE. **a** . Hierarchical heat map cluster showing individual DGE values for TR (restricted diet transgenic) and WT (wild-type) coho salmon. Rows are standardized to have a mean of 0 and standard deviation of 1; red indicates high and green indicates low DGE values. Each rectangle represents individual DGE values **b** . Bar graphs showing DGE values from TR and WT groups (mean ± SE) for *F-box protein 11* (*fbox11*), *E3-ubiquitin ligase Itchya (itchya), F-box and leucine-rich repeat protein 2* (*fbxl2*), *praja ring finger 2 E3 ubiquitin protein ligase* (*praja2*), *ubiquitin protein ligase E3 component n-recognin 4* (*ubr4*), *membrane-associated ring finger* (C*3HC4*) *7 E3 ubiquitin protein ligase* (*march7*), forkhead box K1 (*foxk1*) and forkhead box N4 (*foxn4*). Significant differences are indicated with one (FDR < 0.05) or two asterisk (FDR < 0.01) according to the degree of significance

The mTorc1 complex integrates cues from growth factors to activate ribosome biogenesis and protein synthesis via phosphorylation of ribosomal protein S6 kinase 1 [22]. mTorc1-mediated phosphorylation of the eukaryotic initiation factor 4E (Eif4e) binding protein (4ebp1-3) induces its dissociation from eIF4E and promotes cap-dependent translation [23]. mTorc1 is also an important part of the amino acid sensing machinery in the cell comprising Rags, Ragulator complex and v-Atpase [24]. The amino acid-Rag axis activates mTORC1 through a mechanism involving its translocation to the lysosomal surface [25]. Thus in the absence of sufficient levels of amino acids, the incorrect positioning of mTorc1 renders it insensitive to activation by growth factors [26]. The present transcriptome data provides strong evidence that elevated Gh levels resulted in a widespread increase in translation initiation factor mRNA with 19 out of 44 (43 %) genes significantly higher expressed in TR than WT fish, including *eif4e1* and *eif4e2* (Fig. 4; Additional files 6 and 7). It is clear from the hierarchical cluster of all 44 genes that 70 % of the genes seem to have a higher expression in TR, even if not significant, than WT with only a handful of contigs more expressed in this condition, such as *eif1* (Fig. 4; Additional files 6 and 7). In addition, 14 out of 47 protein chaperones genes (30 %) with roles in protein folding were significantly elevated in TR relative to WT fish, including many with known roles in muscle differentiation and sarcomere assembly such as *unc45* and *hsp90aa1* that are also regulated by nutrition (Fig. 5; Additional file 8) [8, 27]. The majority of E3-ubiquitin ligases analysed, enzymes involved in protein degradation through the Proteasome system, were more highly expressed in WT than TR (however, due to individual variability in WT fish only some of the differences found were significant) (Fig. 6; Additional file 6), which indicates that TR fish were not starving. The differences in translation initiation factors, protein chaperones and E3-ubiquitin ligases expression in TR fish were not matched by a net increased protein accretion since TR and WT fish grew at the same rate [13], implying some increase in the metabolic costs of transcription relative to WT.

### Myoblast fusion

**Fig. 7:**
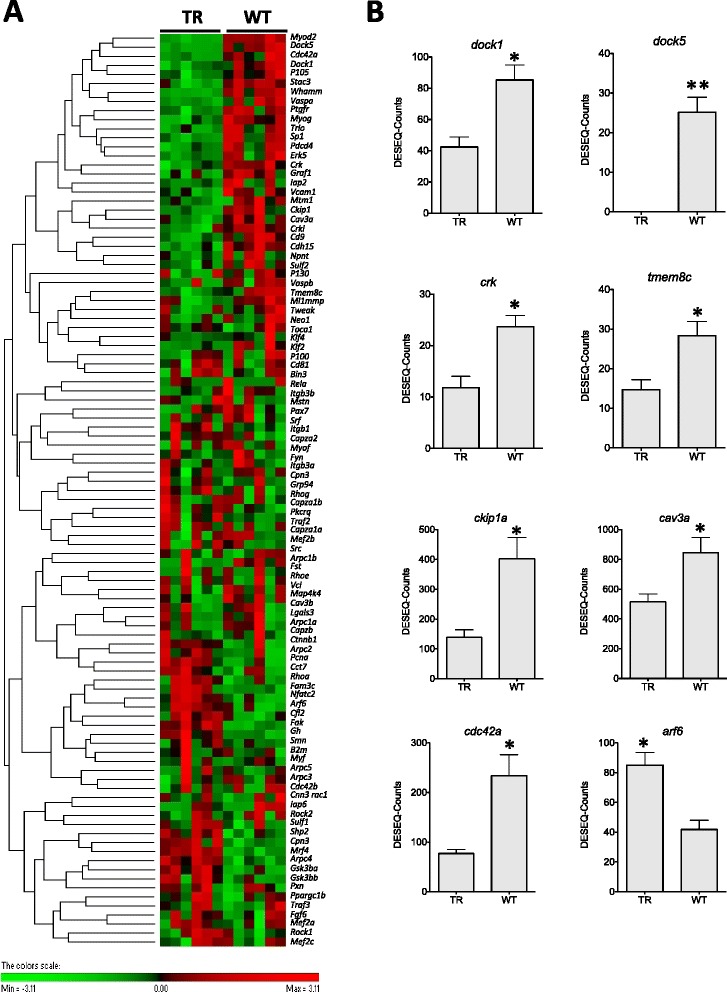
Myoblast fusion related genes DGE. **a** . Hierarchical heat map cluster showing individual DGE values for TR (restricted diet transgenic) and WT (wild-type) coho salmon. Rows are standardized to have a mean of 0 and standard deviation of 1; red indicates high and green indicates low DGE values. Each rectangle represents individual DGE values **b** . Bar graphs showing DGE values from TR and WT groups (mean ± SE) for *dedicator of cytokinesis 1* (*dock1*), *dedicator of cytokinesis 5* (*dock5*), *v-crk avian sarcoma virus CT10 oncogene homolog* (*crk*), *transmembrane protein 8C* (*tmem8c*), *pleckstrin homology domain containing, family O member 1* (*ckip1a*), *caveolin 3a* (*cav3a*). Significant differences are indicated with one (FDR < 0.05) or two asterisk (FDR < 0.01) according to the degree of significance

Myoblast fusion involves multiple sarcolemmal receptors, cell-surface proteins and intracellular signaling pathways leading to membrane and actin cytoskeleton remodelling [28]. Evidence from *in vivo* and *in vitro* studies involving mouse and zebrafish models was used to construct a list of genes required for myoblast fusion and progression. A total of 99 genes including 4R-paralogues (*cav3a, cav3b, cdc42a, cdc42b, cdc37a, cdc37b*) involved in myoblast fusion and differentiation were identified in the transcriptome (Additional file 6). 31 out of the 99 genes (31 %) from the list were differentially expressed between TR and WT groups of coho salmon (Fig. 7; Additional file 9) (FDR < 0.05). A total of 22 of the genes were significantly down regulated in ration-restricted transgenic fish concomitant with a reduction in the number of fast muscle fibres per myotomal cross-section [13]. The majority (10) of these genes (*vaspa*, *cdh15*, *graf1*, *crkl*, *crk*, *dock1*, *trio*, *plekho1a*, *cdc42a* and *dock5*) were associated with signaling through the cell surface protein cadherin (Fig. 7a).

Morpholino antisense-oligonucleotide-mediated knockdown studies have shown that both *dock1* and *dock5* and their adaptor proteins *crk, crk-like* (*crkl*) and the *pleckstrin homology domain containing family member 1a* (*plekho1a* or *ckip1*) are required for the fusion of fast-type myoblasts in zebrafish [29]. Members of the Rho family of guanosine triphosphatases (GTPases), including Rac1, operate downstream of Dock1 to stimulate myoblast fusion [28]. Rac1 is also activated by M-cadherin-dependent adhesion through Trio during C2C12 myoblast fusion [30]. *M-cadherin* (also known as *cadherin15*) and *trio* transcripts were also both significantly downregulated in TR relative to WT salmon (Fig. 7; Additional file 9). On the basis of differences in the defects observed in *dock1*^−/−^ and *trio*^−/−^ mice it has been suggested that Trio is required for myoblast-myoblast fusion, but not for myoblast-myotube fusion [28]. A further three of the genes downregulated in TR fish were associated with integrins and focal adhesion kinase signaling (Fig. 7; Additional file 9). Also down regulated in TR relative to WT fish were *sp1*, a component of Mapk-Erk5 signaling pathway, *nfkb1* which is involved in the non-canonical NF-kB signaling and *tmem8c* or *myomaker* which is required for myoblast-myotube fusion (Fig. 7; Additional file 9) [31]. Nine (*aox1*, *gsk-3ß*, *cpn3*, *arf6, arpc4, shp2, cfl2, mrf4* and *nfatc2*) out of the 99 genes on the list were significantly upregulated in TR relative to WT fish (FDR <0.05) (Fig. 7; Additional file 9). Integrin signaling is mediated by the non-receptor protein kinase Fak promoting myoblast fusion and an associated increase in Caveolin-3 [32], transcripts of which were downregulated in TR relative to WT fish (Fig. 7; Additional file 9). The differences in DGE observed are consistent with a higher myoblast fusion activity in WT when compared with TR, consistent with the observed differences in fibre number [13].

### Quantitative PCR data validation of DGE

**Fig. 8:**
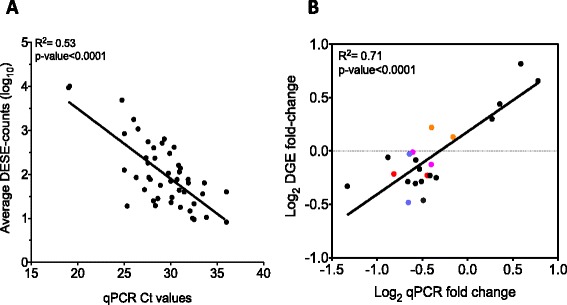
DGE and qPCR correlations. **a**. DGE and qPCR correlations. a. Correlation between DGE and qPCR Ct values for individual genes for each treatment. DGE values are expressed as Log_10_, Pearson R^2^ and P-value for the correlation are indicated in the top-left corner b. Correlation between gene fold-change between TR and WT groups calculated from DGE and qPCR values. Fold-change is expressed in a Log_2_ scale. Pearson R^2^ and P-values for the correlation are indicated in the top-left corner. Circles with different colour than black represent the position of the 4R-paralogues plotted: *cav3a/cav3b* (red circles), *cdc42a/cdc42b* (blue circles), ghra.2/ghrb.1 (pink circles) and *vaspa/vaspb* (orange circles)

In order to validate the results obtained from DGE we analysed the expression of 23 genes covering different ranges of expression (including 3R and 4R-paralogues *cav3a*, *cav3b*, *cdc42a*, *cdc42b*, *vaspa*, *vaspb*, *ghra.2* and *ghrb.1*) by quantitative PCR (qPCR). The qPCR data supports the DGE estimations reported with similar differences between treatments for the individual genes (Additional file 10). Correlation analysis was used to examine the relationship between the levels of expression for individual genes, that were found to be equivalent irrespective of whether the data were expressed as DESEQ-counts and average Ct values (R^2^ = 0.53; *P* < 0.0001) or DGE and qPCR fold-change (R^2^ = 0.71; *P* < 0.0001) (Fig. 8).

### Energy budgeting in the restriction-fed transgenic model

TR fish are more aggressive and exhibit markedly higher levels routine swimming behaviour than WT fish and may have higher metabolic costs associated with the futile expression of mRNAs and proteins required for growth (present study). In spite of these additional costs, TR fish grow at the same rate as WT fish which implies some compensating alterations to energy budgeting. The source of these energy savings includes the cost of ionic homeostasis which has been estimate to contribute 20-40 % of the routine metabolic rate in teleosts [15], a large part of which can be attributable to maintaining the resting membrane potential of fast skeletal muscle which comprises around 70 % of body mass in salmonids. TR fish have fewer, but larger diameter fast muscle fibres than WT for a given body length [13], which will result in reduced ion pumping costs [14, 15]. Our working hypothesis is that the uncoupling of the Gh-axis from energy status directly affects one or more of the signaling pathways regulating myotube formation and hypertrophic growth, and several candidate pathways, particularly signaling through cadherin, have been identified in the present study. Calorie restriction in WT fish may not result in changes in muscle cellularity because the metabolic signals normally influencing endogenous Gh regulation remain intact, a hypothesis requiring further assessment.

## Methods

### Fish

Experiments on coho salmon (*Oncorhynchus kisutch*) were conducted in a non-commercial containment facility for transgenic fish at Fisheries and Oceans Canada, West Vancouver. Wild-type (WT) fish were from the 2010 brood of Chehalis River strain (British Columbia, Canada) [33]. The strain of transgenic coho salmon used (M77) was derived from Chehalis River strain produced using the OnMTGH1 construct comprised of 320 bp of sockeye salmon metallothionein-ß promoter fused to the 5′-UTR region of the full-length type-I growth hormone (*gh1)* gene and the terminator from the same species, as previously described [33]. Fish were reared under standard hatchery conditions in fresh well water (10 ± 1 °C) with a natural photoperiod and fed commercially available salmon diets (Skretting, Vancouver, Canada). The 2010 brood of Gh transgenics (TR group) were fed the same ration to that of the WT group (i.e. pair fed), resulting in similar growth rates in the two groups. Fish were fasted for 24 h prior to humane sacrifice and selected to produce two groups with a similar average fork length (cm) and hence same the developmental stage. All procedures used in the study were approved by the Department of Fisheries and Oceans Pacific Region Animal Care Committee. WT and food-restricted growth hormone transgenic coho salmon (TR) were obtained from the same group used by the authors in a previous study [13]. No significant differences were found in weight or fork length between TR (63.5 ± 3.1 g; 17.4 ± 0.2 cm) and WT (57.7 ± 2 g; 17.1 ± 0.1 cm) used in the present study.

### RNA extraction and sequencing

Sections of pure fast skeletal muscle were carefully dissected from dorsal epaxial myotomes of 6 animals from WT and TR groups matched for body length and with known differences in muscle fibre distribution [13]. Total RNA was extracted by homogenization in 1 ml of Trisure (Bioline, London, UK) using D-Matrix tubes (MP Biomedical, Cambridge, UK) and following the manufacturer’s recommendations. Total RNA concentration, 260/230 and 260/280 ratios were estimated by Nanodrop spectrometer N1000 (Thermo Scientific). RNA integrity was estimated by resolving 1 μg of sample in a 1 % (m/v) ethidium bromide agarose gel. A total of 3 μg of RNA per sample was sent to the Sick-Kids Hospital Next Generation Sequencing service (Vancouver, Canada) for sequencing. Individual barcoded libraries for each animal were paired-end sequenced using two lines of Illumina HiSeq2000. Raw reads were deposited in the EBI-SRA database under the accession number PRJEB7712.

### cDNA synthesis and qPCR reactions

1 μg of total RNA from 6 individuals for each of the treatments (WT and TR) was reverse transcribed to cDNA using the Quantitec reverse transcription kit (QIAGEN, Manchester, UK) including the gDNA wipe-out step to remove remains of genomic DNA and –RT and NT controls were run in parallel with 1 μg of RNA but no RT enzyme or RT enzyme but no template. cDNA samples were diluted 1/50 in Nuclease-free water (QIAGEN). 6 μl per sample were mixed with 7.5 μl of SensiFAST SYBR Lo-ROX 2X master mix (Bioline) containing 400nM of sense/antisense primers. Reactions were performed in duplicated in a Mx3005P thermocycler (Agilent, Oxford, UK), with 1 cycle of 2 min at 95 °C and x40 cycles of 5 s 95 °c and 20s at 65 °C, followed by a dissociation curve analysis, where a single peak was detected in all cases.

Primers were designed using Primer 3 [http://biotools.umassmed.edu/bioapps/primer3_www.cgi] to amplify products between 100-200 bp from gene sequences retrieved from a *de novo* coho salmon skeletal muscle transcriptome (primers and sequences used for primer design can be found in Additional file 11). Netprimer (PremierBiosoft) [http://www.premierbiosoft.com/netprimer/] was used to detect primer hairpins and cross-dimmers. When two salmonid paralogues were identified primers were design to bind to the most divergent regions of sequence. Genorm software [34] was used to evaluate the stability of the four reference genes analysed *rpl27*, *rpl13*, *ef1a* and *ßactin*. The *rpl13* and *ef1a* were found to be the most stable reference genes (M = 0.058). Normalization of gene expression was performed using the geometric average of *rpl13* and *ef1a*. All expression values are expressed as arbitrary units.

### De novo transcriptome assembly and annotation

Quality filtered raw reads were *de novo* assembled by Sick-Kids Bioinformatic services (Vancouver, Canada) using Trinity software [35] (the complete *de novo* assembly can be found in the Additional files 12, 13, 14, 15 and 16). Resulting contigs were identified by BLAST (BLASTx) against the NCBI non-redundant database (nr) and Gene Ontology (GO) annotated using Blast2GO software default settings [36]. In order to identify the number of unique genes BLASTx results were manually investigated to remove duplicates and those contigs annotated as hypothetical proteins or predicted proteins.

Fully annotated contigs (with positive BLAST and GO results) were BLAST against the KEGG collection of metabolic and signaling pathways with the KEGG Automatic Annotation Service (KAAS) using the single-directional best hit (SBH) method against all vertebrates pathways deposited in the database [37]. For identification of coding sequences in the contigs generated during the *de novo* assembly the complete zebrafish proteome [38] was blasted (tBLASTn) against all annotated contigs using BioEdit software [www.mbio.ncsu.edu/bioedit/bioedit.html]. Alignment data from positive hits results between coho salmon contigs and zebrafish gene amino acid sequences gave us an estimation of the percentage of coding sequences contained in the *de novo* contigs.

### Digital gene expression 

Read mapping, read normalization and global digital expression were carried out by the bioinformatics department of Sick-Kids Hospital, Next Generation Sequencing service (Vancouver, Canada). Quality filtered raw reads from individual libraries were mapped to the complete *de novo* coho salmon transcriptome and their abundance estimated using the RSEM [39]. DESEQ program from the R-Bioconductor package was used to estimate global differences in digital gene expression (DGE) between TR and WT groups [40]. The DESEQ program normalizes mapped reads for individual samples by contig length and library depth using a negative binomial distribution previous to test differences in reads mapped between conditions. To test specific hypothesis for particular physiological processes DESEQ normalized counts (DESEQ-counts) per individual animal were retrieved.

### Gene ontology (GO) enrichment analysis

GO enrichment analysis from those genes differently expressed between TR and WT after the global DGE analysis was performed using the STRING webserver [41]. In order to maximize GO enrichment analysis BLAST results were manually annotated with their human orthologue abbreviated gene name (e.g. contig identified as *dedicator of cytokinesis protein 5* was named as *DOCK5*).

### Paralogue identification and phylogenetic analysis

A phylogenetic analysis was carried to confirm the salmonid-specific WGD origin of potential 4R paralogues in the transcriptome. Potential 4R-paralogues were conceptually translated to their amino acid sequence. BLASTp against the non-redundant NCBI database was used to confirm the identities of the translated paralogues. Teleost orthologues for the genes of interest were retrieved from Ensembl including human and mouse orthologues to be used as outgroups. Potential coho salmon 4R paralogues were used as a query against the rainbow trout (*Oncorhynchus mykiss*) protein collection (BLASTp) and the Atlantic salmon (*Salmo salar*) Transcriptome Shotgun Assembly (TSA) data deposited in the NCBI (tBLASTn). Positive BLAST hits were included in the analysis. Amino acids sequences were aligned using Guidance webserver [42] with MAFFT as MSA algorithm. Only aligned sections with a score over 0.93 were used to generate the phylogenetic tree. Evolutionary models were estimated for all alignments using MEGA5 [43]. In all cases the best evolutionary model was estimated to be JTT + G (data not show). Finally Maximum likelihood trees for each alignment were constructed using PhyML webserver [44] and displayed using FigTree [http://tree.bio.ed.ac.uk/software/figtree].

### Statistical analysis

For global DGE between TR and WT individuals the DESEQ program was used following programmer recommendations and a false discovery rate (FDR) cut-off of FDR < 0.05 was applied for significant differences. The original list of genes differently expressed generated by DESEQ was manually curated and only those contigs with >15 DESEQ-counts and over 2log_2_ fold-change were used. As expected from previous studies [45, 46], coho salmon *de novo* transcriptome presented a significant degree of redundancy; therefore, more than one contig shared the same BLAST results (“sister” contigs). The original DESEQ list of genes was further curated, maintaining those genes that showed consistent expression between “sister contigs”, special care was taken to detect 3R and 4R-paralogues annotated with the same ID by investigating the alignment of sister contigs.

Differences in expression for specific groups of genes were estimated using DESEQ-counts values per individual animal for each gene (*n* = 6 for each experimental group). Significant differences were detected by a two-tailed test between WT and TR individuals followed by a Benjamin-Hochberg (FDR) correction for multiple comparisons. Significant differences were accepted when FDR < 0.05.

Hierarchical clustering analysis of gene expression data was performed using Permutmatrix with gene expression normalized by row using McQuitty’s method [47]. Principal component analysis for gene expression of the 12 coho salmon analysed was performed using PASW Statistic software v21 (IBM).

Differences in gene expression analysed by qPCR were analysed by t-test and a significant threshold of *P* < 0.05. Pearson correlation was used to study qPCR and DGE expression data obtained for the same genes. In order to facilitate visualization of results data was logarithmically transformed (Log_10_ for expression data and Log_2_ for fold-change data) prior to correlation analysis. Pearson correlation was estimated using PASW Statistic software v21.

## Conclusions

Gh-transgenic coho salmon show increased appetite and growth relative to wild type fish. We restricted the food intake of transgenic fish (TR) to that of wild-type (WT) fish fed to satiation, resulting in higher levels of muscle Gh expression, but a similar final body size. The two groups had markedly different gene expression signatures, with TR fish showing increased transcript abundance for pathways associated with protein translation and protein folding and reduced expression of genes involved with myotube fusion. The down-regulation of genes with known function in myoblast fusion, particularly cadherin signaling, was correlated with a reduction in average muscle fibre diameter in TR relative to WT fish which is expected to reduce the costs of maintaining ionic homeostasis. This may explain why TR fish are more active than WT fish yet grow at a similar rate.

## Availability of supporting data

All supporting data for the present manuscript is included as additional files.

## Additional files

Additional file 1:
**Annotated transcriptome sequences (.zip), sequences were annotated using BLAS2GO (BLASTx) against the NCBI non-redundant database (nr) unigene IDs present in the transcriptome (.txt). KAAS file containing mapping results (.hier) and the transcriptome was annotated against all vertebrate pathways present in the KAAS online server. **(ZIP 13705 kb) Additional file 2:
**Gene sequences containing >90% of the coding sequence (.fasta). **Coding sequence coverage distribution from annotated unigenes present in the coho salmon transcriptome (shaded area represents those contigs containing 100 to 50% of the coding sequence). (PDF 316 kb) Additional file 3:
**Paired-end reads generated for each individual library and the percentage of assembly**. (XLSX 54 kb) Additional file 4:
**Genes with significant changes in their expression between restriction-fed growth hormone-transgenic coho salmon (TR) and wild-type salmon (WT) after DESEQ analysis of the transcriptome as a whole (.excel).** (XLSX 61 kb) Additional file 5:
**Maximum-likelihood phylogenetic trees, genes sequences and alignments used to resolve 4R-salmonid paralogues found during the study.** (ZIP 4 kb) Additional file 6:
**Genes related to myoblast fusion, the Gh-Igf network, chaperones/co-chaperones, initiation factors, E3-ubiquitin ligases, initiation factors and autophagy, retrieved from the transcriptome to test specific hypothesis.** WT 1–5 and TR 1–5 values represent DESEQ-counts for individual animals. (XLSX 171 kb) Additional file 7:
**DGE results of genes involved with initiation of protein translation.** (DOCX 102 kb) Additional file 8:
**DGE results of genes involved with protein folding.** (DOCX 79 kb) Additional file 9:
**DGE results of genes involved with myoblast fusion.** (DOCX 105 kb) Additional file 10:
**Bar graphs of qPCR results for individual genes. Gene expression is expressed in arbitrary units (a.u).** Significant differences between groups are indicated with an asterisk (*P* < 0.05). (PDF 402 kb) Additional file 11:
**Quantitative PCR primer sequences, PCR efficiencies and melting temperatures for genes analysed**. (DOCX 143 kb) Additional file 12:
***de novo*** **coho salmon transcriptome (part 1 of 5)**. (ZIP 14691 kb) Additional file 13:
***de novo*** **coho salmon transcriptome (part 2 of 5).** (ZIP 10798 kb) Additional file 14:
***de novo*** **coho salmon transcriptome (part 3 of 5).** (ZIP 9435 kb) Additional file 15:
***de novo*** **coho salmon transcriptome (part 4 of 5).** (ZIP 5145 kb) Additional file 16:
***de novo*** **coho salmon transcriptome (part 5 of 5).** (ZIP 8207 kb)
