# Supplementary figures and images for: RNAseq analysis of fast skeletal muscle in restriction-fed transgenic coho salmon (Oncorhynchus kisutch): an experimental model uncoupling the growth hormone and nutritional signals regulating growth
Source: BMC Genomics. 2015 Jul 31;16(1):564. doi: 10.1186/s12864-015-1782-z (PMC4521378; doi:10.1186/s12864-015-1782-z)

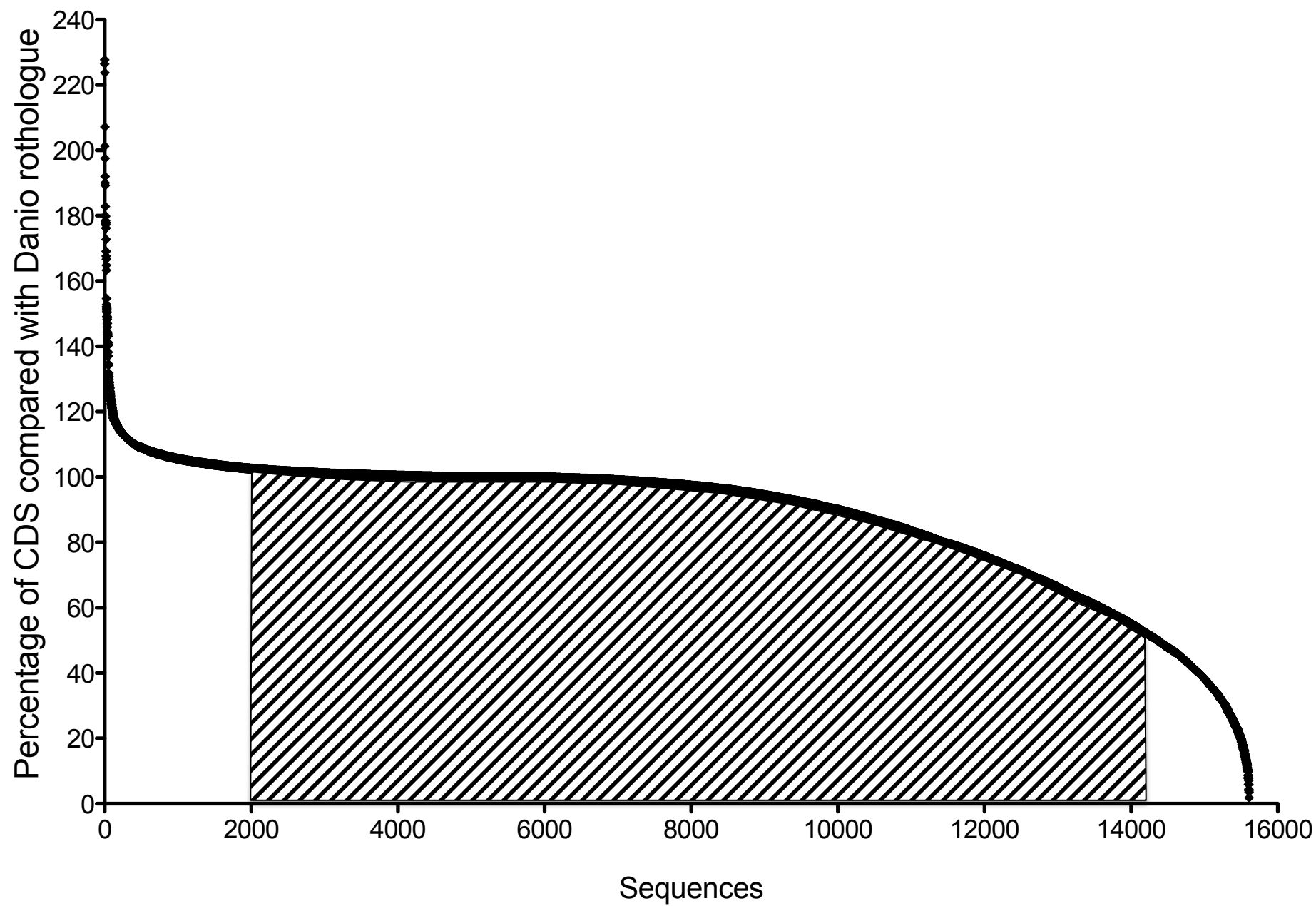

Supplement: Additional file 2: — Gene sequences containing >90% of the coding sequence (.fasta). Coding sequence coverage distribution from annotated unigenes present in the coho salmon transcriptome (shaded area represents those contigs containing 100 to 50% of the coding sequence). (PDF 316 kb) [file 12864_2015_1782_MOESM2_ESM.pdf]

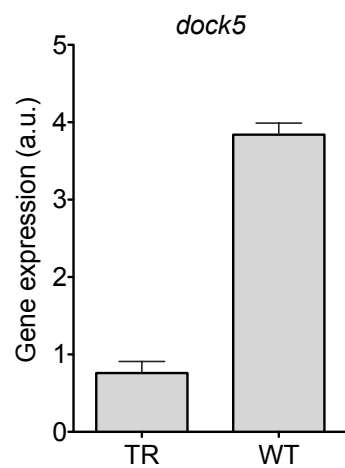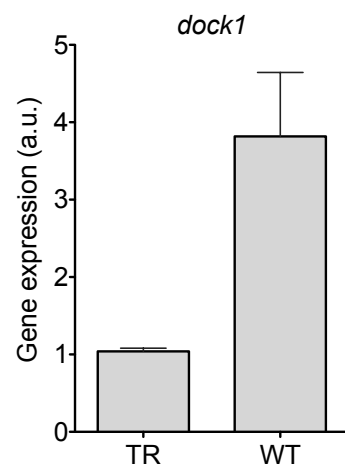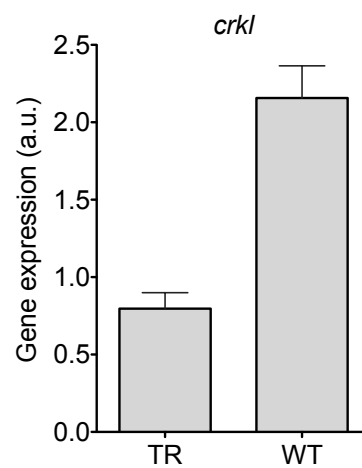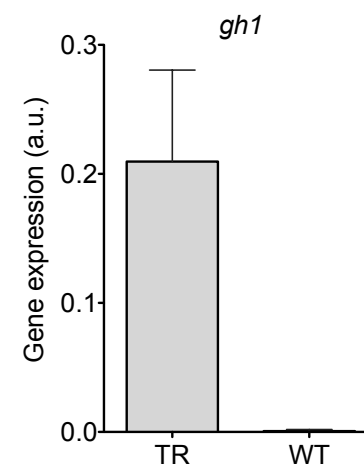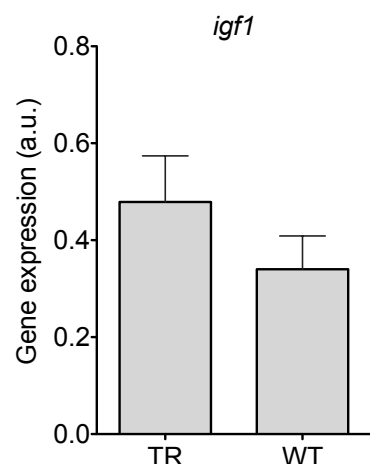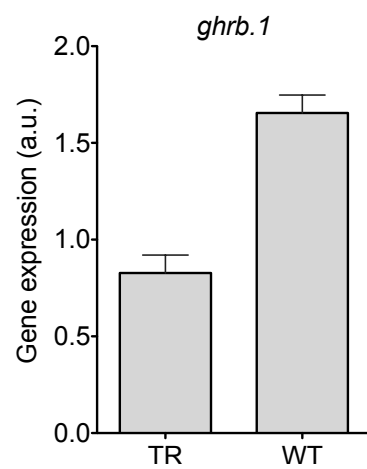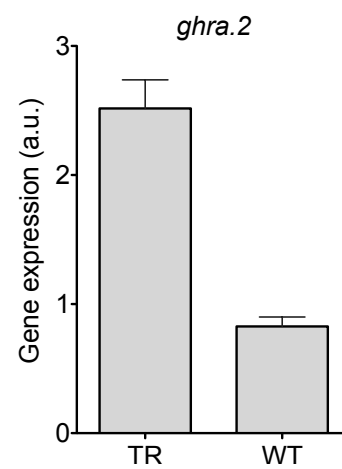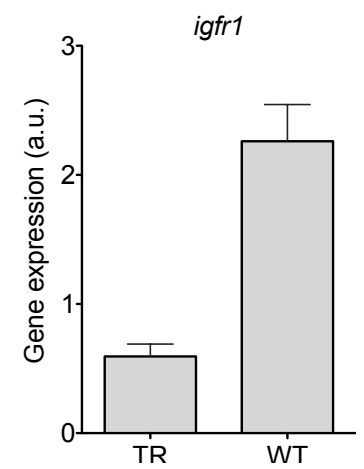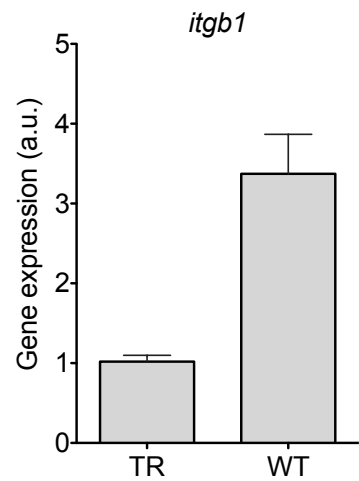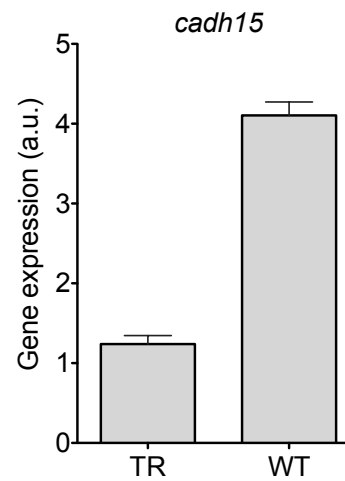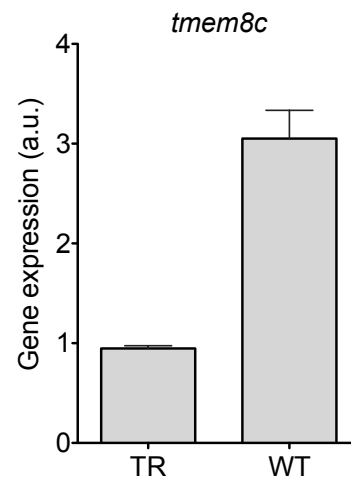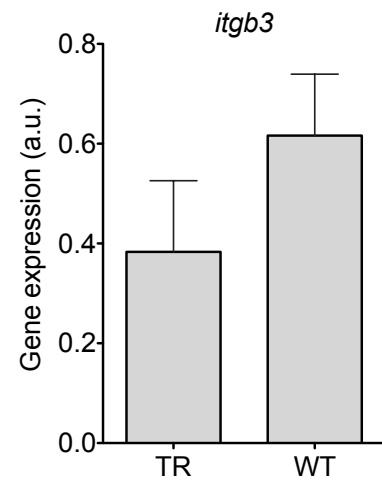

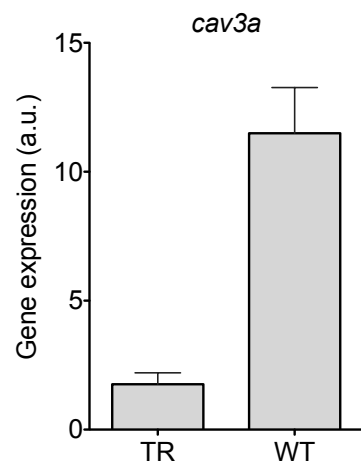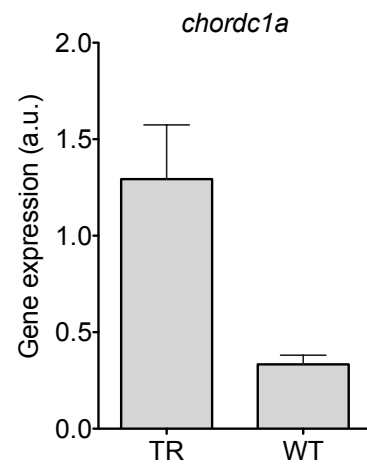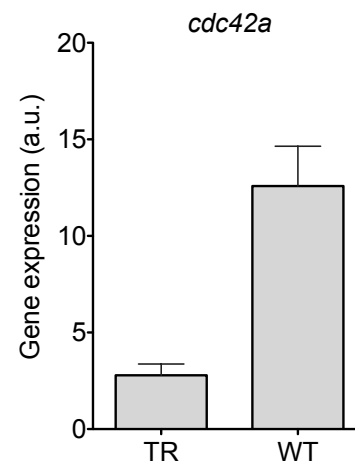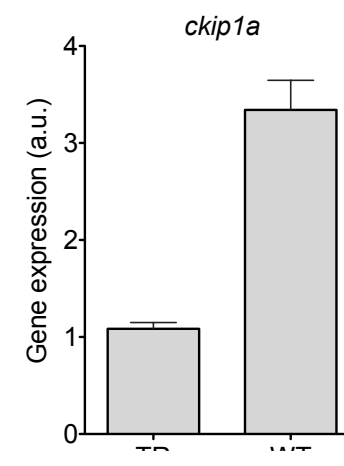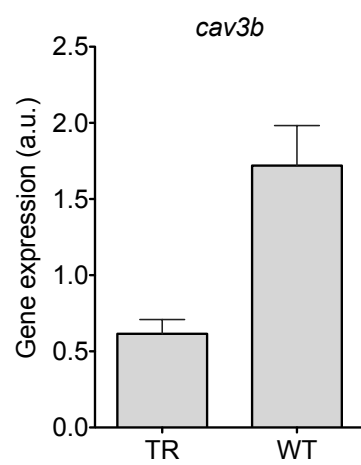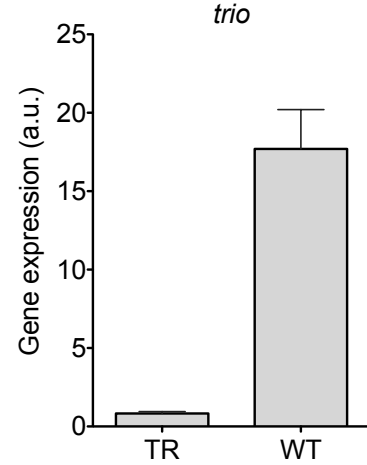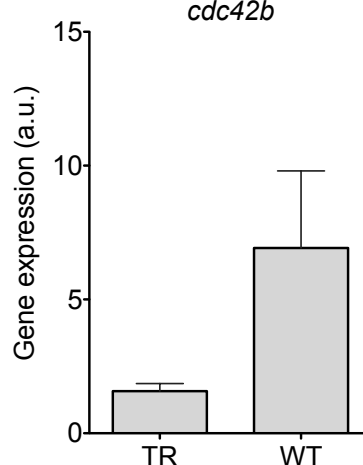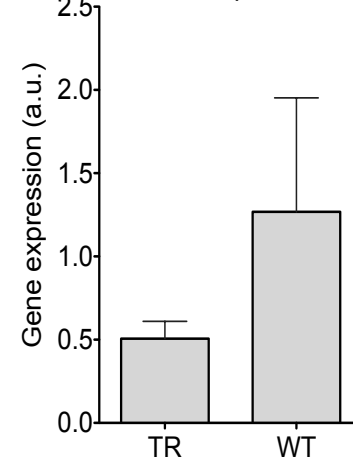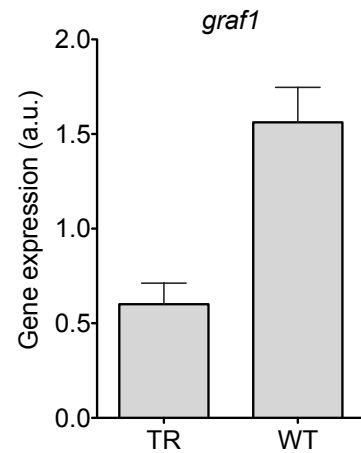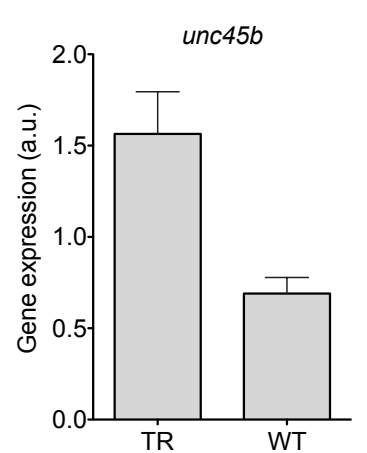

Supplement: Additional file 10: — Bar graphs of qPCR results for individual genes. Gene expression is expressed in arbitrary units (a.u). Significant differences between groups are indicated with an asterisk (P < 0.05). (PDF 402 kb) [file 12864_2015_1782_MOESM10_ESM.pdf]
