# Supplementary material for: RNAseq analysis of fast skeletal muscle in restriction-fed transgenic coho salmon (Oncorhynchus kisutch): an experimental model uncoupling the growth hormone and nutritional signals regulating growth
Source: BMC Genomics. 2015 Jul 31;16(1):564. doi: 10.1186/s12864-015-1782-z (PMC4521378; doi:10.1186/s12864-015-1782-z)
Supplement: Additional file 7: — DGE results of genes involved with initiation of protein translation. (DOCX 102 kb) [file 12864_2015_1782_MOESM7_ESM.docx]

Genes involved with initiation of protein translation.

TR: restriction-fed growth hormone-transgenic; WT: wild-type; Counts: Average DESEQ-counts (Mean ± SE); FDR: False discovery rate.

| Gene name | ZFIN  ID | TR  Counts | WT  Counts | Ratio | FDR |
| --- | --- | --- | --- | --- | --- |
| Eukaryotic translation initiation factor 4E1C | *eif4e1c* | 329±59 | 42±7 | 7.7 | 0.004 |
| Eukaryotic translation initiation factor 5B | *eif5b* | 806±124 | 204±63 | 3.9 | 0.007 |
| Eukaryotic translation initiation factor 5 | *eif5* | 2790±194 | 733±144 | 3.8 | 0.0003 |
| Eukaryotic translation initiation factor 2D | *eif2d* | 118±12 | 40±10 | 2.9 | 0.004 |
| Eukaryotic translation initiation factor 3 subunit B | *eif3b* | 3084±365 | 1497±302 | 2.0 | 0.01 |
| Eukaryotic translation initiation factor 3J | *eif3j* | 119±10 | 57±6 | 2.0 | 0.003 |
| Eukaryotic translation initiation factor 2 subunit 1 | *eif2s1* | 365±51 | 179±17 | 2.0 | 0.01 |
| Eukaryotic translation initiation factor 4 gamma 2 | *eif4g2* | 151±17 | 79±13 | 1.9 | 0.01 |
| Eukaryotic translation initiation factor 3K | *eif3k* | 382±26 | 210±14 | 1.8 | 0.001 |
| Eukaryotic translation initiation factor E4 | *eif4e* | 294±26 | 160±18 | 1.8 | 0.008 |
| Eukaryotic translation initiation factor 3F | *eif3f* | 134±5 | 80±7 | 1.7 | 0.001 |
| Eukaryotic translation initiation factor 4H | *eif4h* | 2130±97 | 1348±77 | 1.6 | 0.001 |
| Eukaryotic translation initiation factor 6 | *eif6* | 250±15 | 169±18 | 1.4 | 0.01 |
| Eukaryotic translation initiation factor 2-alpha kinase 1 | *eif2ak1* | 353±46 | 520±41 | 0.7 | 0.04 |
| Eukaryotic translation initiation factor 4 subunit G3 | *eif4g3* | 104±9 | 147±7 | 0.7 | 0.01 |
| Eukaryotic translation initiation factor 4B | *eif4b* | 342±44 | 560±55 | 0.6 | 0.02 |
| Eukaryotic translation initiation factor 1B | *eif1b* | 1997±172 | 3585±198 | 0.5 | 0.001 |
| Eukaryotic translation initiation factor 4 binding protein 1 | *eif4ebp1* | 586±58 | 1429±129 | 0.4 | 0.001 |
| Eukaryotic translation initiation factor 4E3 | *eif4e3* | 130±14 | 490±94 | 0.2 | 0.01 |
