# Supplementary material for: RNAseq analysis of fast skeletal muscle in restriction-fed transgenic coho salmon (Oncorhynchus kisutch): an experimental model uncoupling the growth hormone and nutritional signals regulating growth
Source: BMC Genomics. 2015 Jul 31;16(1):564. doi: 10.1186/s12864-015-1782-z (PMC4521378; doi:10.1186/s12864-015-1782-z)
Supplement: Additional file 8: — DGE results of genes involved with protein folding. (DOCX 79 kb) [file 12864_2015_1782_MOESM8_ESM.docx]

Molecular chaperones and co-chaperones.

TR: restriction-fed growth hormone-transgenic coho salmon; WT: wild-type coho salmon; Counts: DESEQ-Normalised number of reads mapped Mean ± SE; FDR: False discover rate.

| Gene name | ZFIN  ID | TR  Counts | NT  Counts | Ratio | FDR | Remarks |
| --- | --- | --- | --- | --- | --- | --- |
| Heat shock protein 90 alpha 1 | *hsp90aa1* | 7668±1288 | 1120±265 | 6.8 | 0.005 | Salmonid paralogues Hsp90a.1a and 1.b increase their expression during myotube differentiation [8; 27]. |
| Cysteine histidine-rich-domain containing 1b | *chordc1b* | 125±11 | 19±4 | 6.5 | 0.02 | Inhibits kinase activity of ROCK2 [55] and important kinase involved in myogenesis [52]. |
| Heat shock protein 75, TNF receptor-associated protein1 | *trap1* | 137±19 | 33±11 | 4.1 | 0.005 | Mitochondrial chaperone involved in heart hypertrophy [53]. |
| Heat shock protein 10 | *hspe1* | 432±26 | 120±29 | 3.6 | 0.0003 | Increase protein abundance in the early stages of C2C12 differentiation [54]. |
| Unc-45 homologB | *unc45b* | 4894±330 | 1777±555 | 2.7 | 0.03 | Hsp90 co-chaperone essential for sarcomere assembly in zebrafish [27] and may have a role in myoblast fusion in C2C12 cell line [55]. |
| Heat shock protein 60 protein 1 | *hspd1* | 1477±159 | 579±205 | 2.5 | 0.02 | Interacts with atrogin-1 in response to myostatin [56]. Also increase expression in myobutes with disorganized cytoskeleton [57]. |
| Prostaglandin-E Synthase 3b | *ptges3b* | 148±16 | 59±14 | 2.5 | 0.008 | Chaperone that increase its expression in response to single meal intake in zebrafish skeletal muscle [56]. |
| Heat shock protein 4 | *hspa4* | 215±18 | 98±14 | 2.1 | 0.005 | Expression reduced at protein and transcript level in pigs with critical illness myopathy [57]. |
| Cysteine histidine-rich-domain containing 1 | *chordc1a* | 2664±304 | 1283±129 | 2.0 | 0.0003 | Inhibits kinase activity of ROCK2 [51] and important kinase involved in myogenesis [52]. |
| Stress induced phosphoprotein 1 | *stip1* | 2566±170 | 1470±115 | 1.7 | 0.005 | Identified in both undifferentiated and differentiated C2C12 [58]. Upregulated after a single meal in zebrafish skeletal muscle [59]. |
| Prostaglandin-E Synthase 3a | *ptges3a* | 960±114 | 596±54 | 1.6 | 0.03 | Chaperone that increase its expression in response to single meal intake in zebrafish skeletal muscle [56]. |
| Cell division cycle 37 | *cdc37* | 263±9 | 178±17 | 1.4 | 0.008 | Hsp90 co-chaperone that mediates interaction with MyoD and Fyn. When cdc37-hsp90 is blocked myoblast differentiation fails [60]. |
| Heat shock protein 27 | *hspb1* | 375±39 | 676±60 | 0.5 | 0.008 | Inhibition of hspb1 reduces fibre growth in zebrafish cranio-facial muscles [61]. |
| Aryl Hydrocarbon Receptor Interacting Protein | *aip* | 29±6 | 73±8 | 0.4 | 0.008 | Hsp90 co-chaperone involved in AhR receptor [62]. Deletion of both IAP copies is embryonically lethal due to serious abnormalities [63]. |

8. Garcia de la serrana D, Johnston IA: **Expression of heat shock protein 90 (Hsp90) paralogues is regulated by amino acids in skeletal muscle of Atlantic salmon.** *PLoS ONE* 2013, 8**:**e74295.

27. Du J, Bernick E: **Molecular chaperones Hsp90a1 and Unc45b are required for sarcomere assembly in skeletal muscles of zebrafish embryos**. *FASEB* 2011, **28**:10.

52. Pelosi M, et al: **ROCK2 and its alternative splice isoform ROCK2m positively control the maturation of the myogenic program**. *Mol Cel Biol* 2007, **27**:6163-176.

53. Zhang Y, Jiang DS, Yan L, Cheng KJ, Bian ZY, Lin GS: **Hsp75 protects against cardiac hypertrophy and fibrosis**. *J Cell Biochem* 2011, **112**:1787-94.

54. Casadei L, Vallorani L, Gioacchini AM, et al: **Proteomics-based investigation in C2C12 myoblast differentiation**. *Eur J Histochem* 2009, **53**:261-8.

55. Price MC, Landsverk ML, Barral JM, Epstein HF: **Two mammalian UNC-45 isoforms are related to distinct cytoskeletal and muscle-specific functions**. *J Cell Scien* 2002, **115**:4013-4023.

56. Amaral IPG, Johnston IA: **Insulin-like growth factor (IGF) signalling and genome wide transcriptional regulation in fast muscle of zebrafish following a single-satiating meal.** *J Exp Biol* 2010, **214**:2125-2139.

57. Lokireddy S, et al: **Identification of atrogin-1 targeted proteins during myostatin-induced skeletal muscle wasting.** *Am J Physiol Cell Physiol* 2012, **303**:512-529.

58. Banduseela VC, Chen YW, Kultima HG, Norman HS, Aare S, Radell P, Eriksson LI, Hoffman EP, Larsson L: **Impaired autophagy, chaperone expression, and protein synthesis in response to critical illness interventions in porcine skeletal muscle**. *Physiol Genomics* 2013, **45**:477-486.

59. Tassin A, Leroy B, et al: **FSHD Myotubes with Different Phenotypes Exhibit Distinct Proteomes**. *PLoS ONE* 2012, **7**:e51865.

60. Lawrence GP, Carrière JF, Kelly JF, Megeney LA: **Comparative analysis of phosphoprotein-enriched myocyte proteomes reveals widespread alteration during differentiation.** *FEBS Letters* 2004, **574**:138-144.

61. Yun BG, Matts RL: **Differential effects of Hsp90 inhibition on protein kinases regulating signal transduction pathways required for myoblast differentiation.** *Exp Cell Res* 2005, **307**:212-223.

62. Middleton RC, Shelden EA: **Small heat shock protein HSPB1 regulates growth of embryonic zebrafish craniofacial muscles**. *Exp Cell Res* 2013, **319**:860-874.

63. Meyer BK, Perdew GH: **Characterization of the AhR-Hsp90-XAP2 core complex and the role of the Immunophilin-related protein XAP2 in AhR stabilization**. *Biochem* 1999, **38**:8907-17.
