# Supplementary material for: RNAseq analysis of fast skeletal muscle in restriction-fed transgenic coho salmon (Oncorhynchus kisutch): an experimental model uncoupling the growth hormone and nutritional signals regulating growth
Source: BMC Genomics. 2015 Jul 31;16(1):564. doi: 10.1186/s12864-015-1782-z (PMC4521378; doi:10.1186/s12864-015-1782-z)
Supplement: Additional file 9: — DGE results of genes involved with myoblast fusion. (DOCX 105 kb) [file 12864_2015_1782_MOESM9_ESM.docx]

Genes involved with myotube formation and hypertrophic growth.

TR: restriction-fed growth hormone-transgenic coho salmon; WT: wild-type coho salmon; Counts: DESEQ-Normalised number of reads mapped Mean ± SE; FDR: False discover rate; Inf: infinte.

| Gene | ZFIN ID | TR  Counts | WT Counts | Ratio | FDR | Remarks |
| --- | --- | --- | --- | --- | --- | --- |
| Aldehyde oxidase | *aox1* | 127±16 | 11±11 | 11.2 | 0.043 | siRNA knockdown in C2C12 cells reduces myotube formation and myogenin protein expression [64]. |
| Glycogen synthase kinase-3a | *gsk-3ß* | 61.9±8 | 21.7±6 | 2.9 | 0.025 | Component classical Wnt signaling pathway. Inactivation leads to stabilisation and nuclear transport of B-catenin promoting mouse myoblast fusion [65]. |
| ADP-Ribsoylation Factor 6 | *arf6* | 85.1±8 | 41.8±6 | 2.0 | 0.025 | *Arf6* silencing inhibits the association of Trio and Rac1 with cadherin impairing mouse myoblast fusion [66]. |
| Nuclear factor of activated T-cells, cytoplasmic 2 | *nfatc2* | 26±4 | 14±1 | 1.9 | 0.037 | Nfatc2^-/-^ knockout mice have smaller myotubes with fewer myonuclei-phenotype rescued with overexpression [67]. |
| Vasodilator-stimulated phosphoprotein | *vaspa* | 106.4±10 | 63.9±3 | 1.7 | 0.025 | siRNA-mediated knockdown in MDCK cells impairs cell-matrix adhesion and migration [68]. |
| Protein tyrosine kinase 2b (Focal Adhesion Kinase, FAK) | *ptk2.2* | 51.5±5 | 33.5±4 | 1.5 | 0.043 | FAK-signaling regulates Caveolin 3 and 1 Integrin  Expression required for myoblast fusion [28]. |
| m-Cadherin | *cdh15* | 161±10 | 237±15 | 0.68 | 0.025 | Cell adhesion molecule with essential role in myoblast fusion^4^, activates Rac-1 expression [69]. |
| CD9 antigen (p24) | *cd9* | 184±12 | 280±24 | 0.65 | 0.005 | Transmembrane 4 superfamily  (TM4SF) protein, associates with -integrins and promotes myoblast fusion [70]. |
| Caveloin-3a | *cav3* | 515±53 | 844±102 | 0.61 | 0.048 | FAK-mediated signalling increases caveolin-3 and 1D-integrin protein levels promoting myoblast fusion [28]. |
| Rho GTPase activating protein 26 (ARHGAP26)(=GRAF1) | *graf1* | 11±2 | 18±2 | 0.59 | 0.037 | GRAF1-deficient mice show reduced myoblast fusion [71] |
| Sp1 transcription factor | *sp1* | 172±13 | 302±31 | 0.57 | 0.025 | Component of MAPK signaling pathway regulating myoblast fusion. Sp1 is activated by ERK5 which in turn activates Kruppel-like factor 2 (klf2) and klf4 [72] |
| v-crk sarcoma virus CT10 oncogene homolog (avian)-like | *crkl* | 64±13 | 113±9 | 0.56 | 0.043 | Adaptor proteins that physically interact with Dock proteins. Morpholino knockdown in zebrafish embryos inhibits myoblast fusion whereas verexpression enhances fusion [29]. |
| Transmembrane protein 8C | *tmem8c* | 15±2 | 28±4 | 0.52 | 0.037 | Transiently expressed on the surface of mouse myoblasts and required for fusion [31]. |
| V-crk sarcoma virus CT10 oncogene homolog (avian) | *crk* | 12±2 | 24±2 | 0.50 | 0.025 | As for crkl above [29]. |
| Dedicator of cytokinesis 1 | *dock1* | 42±6 | 85±10 | 0.50 | 0.0025 | Dock1^-/-^ mice show defect in primary myoblast fusion [73]. Zebrafish morpholino knockdown dock1 reduces myoblast fusion [29]. |
| Nuclear factor of kappa light polypeptide gene enhancer in B-cells 1(p105) | *nfkb1* | 18.9±4 | 39.3±5 | 0.48 | 0.037 | TWEAK and cIAP1 regulate myoblast fusion through the noncanonical NF- signaling pathway [74]. |
| Trio Rho guanine nucleotide exchange factor | *trio* | 36±5 | 77±11 | 0.47 | 0.034 | M-cadherin-dependent adhesion activates Rac1 through Trio in C2C12 myoblast fusion [30]. |
| Pleckstrin homology domain containing, family O member 1a | *plekho1a/ckip1* | 139±26 | 401±72 | 0.34 | 0.032 | Plekho1a (CKIP-1a) knockdown impairs myoblast fusion in mammalian cells and zebrafish embryos [75]. |
| Cell division cycle 42 | *cdc42a* | 77±8 | 234±43 | 0.33 | 0.028 | Rho GTPase family –Rac1 and cdc42 stimulate mouse myoblast fusion *in vivo* and in vitro [76]. |
| Dedicator of cytokinesis 5 | *dock5* | 0 | 25±4 | Inf | 0.003 | Dock5 knockdown in zebrafish embryos reduces myoblast fusion [28]. Dock5^-/-^ mice show normal muscle development, but Dock1^-/-^Dock5^+/-^ mice exhibit mononuclear myotubes and elongation defects [73]. |

28. Hindi SM, Marjan M, Kumar A: **Signaling Mechanisms in Mammalian Myoblast Fusion**. *Sci Signal* 2013, **6**:272.

29. Moore CA, Parkin CA, Bidet Y, Ingham PW: **A role for the Myoblast city homologues Dock1 and Dock5 and the adaptors Crk and Crk-like in zebrafish myoblast fusion**. *Develop* 2007, **134**:3145-3153.

30. Charrasse S, Comunale F, Grumbach Y, Poulat F, Blangy A, Gauthier-Rouviére C: **RhoA GTPase regulates m-cahderin activity and myoblast fusion**. *Mol Biol Cell* 2006, **17**:749-759.

31. Millay DP, O’Rourke JR, Sutherland LB, Bezprozvannya S, Shelton JM, Bassel-Duby R, Olson EN: **Myomaker is a membrane activator of myoblast fusion and muscle formation**. *Nature* 2013, **499**:301-305.

64. Kamli MR, Kim J, Pokharel S, Jan AT, Lee EU, Choi I: **Expressional studies of the aldehyde (AOX1) gene during myogenic differentiation in C2C12 cells**. *Biochem Biophys Res Comm* 2014, **450**:1291-1296.

65. Lin BC, Sullivan R, Lee Y, Moran S, Glover E, Bradfield CA: **Deletion of the aryl hydrocarbon receptor-associated protein 9 leads to cardiac malformation and embryonic lethality.** *J Biol Chem* 2007, **282**:35924-32.

66. Bach AS, Enjalbert S, Comunale F, Bodin S, Vitale N, Charrasse S, Gauthier-Rouvière C: **ADP-ribosylation factor 6 regulates mammalian myoblast fusion through phospholipase D1 and phosphatidylinositol 4,5-biphosphate signalling pathways**. *Mol Biol Cell* 2010, **21**:2412-2424.

67. Horsley V, Friday BB, Matterson S, Kegley KM, Gephart J, Pavlath GK: **Regulation of growth of multinucleated muscle cells by an NFATC2-dependent pathway**. *J Cell Bio*l 2001, **153**:329-338.

68. Zhang Y, Tu Y, Gkretsi V, Wu C: **Migfilin interacts with vasodilator-stimulated phosphoprotein (VASP) and regulates VASP localization to cell matrix adhesions and migration**. J Biol Chem 2006, 281:12397-12407.

69. Krauss RS: **Regulation of promygenic signal transduction by cell-cell contact and adhesion**. *Exp Cell Res* 2010, **316**:3042-3049.

70. Tachibana I, Hemler ME: **Role of transmembrane 4 superfamily (TM4SF) proteins CD9 and CD81 in muscle cell fusion and myotube maintenance**. *J Cell Bio* 1999, **146**:893-904.

71. Lenhart KC, Becherer AL, Li J, Xiao X, McNally EM, Mack CP, Taylor JM: **GRAF1 promotes ferlin-dependent myoblast fusion.** *Dev Biol* 2014, **393**:298-311.

72. Sunadome K, Yamamoto T, Ebisuya M, Kondoh K, Sehara-Fujisawa A, Nishida E: **ERK5 regulates muscle cell fusion through klf transcription factors**. *Dev Cell* 2011, **20**:192-205.

73. Laurin M, Fradet N, Blangy A, Hall A, Vuori K, Coté JF: **The atypical Rac activator Dock180 (Dock1) regulates myoblast fusion in vivo**. *Proc Natl Acad Sci* 2008, **105**:15446-15451.

74. Enwere EK, Holbrook J, Lejmi-Mrad R, Vineham J, Timusk K, Sivaraj B, Issac M, Uehling D, Al-awar R, LaCasse E, Korneluk RG: **TWEAK and cIAP1 regulate myoblast fusion through the noncanonical NF-kB signalling pathway**. *Sci Signa*l 2012, **16** doi:10.1126/scisignal.2003086.

75. Baas D, Caussanel-Boude S, Guiraud A, Calhabeu F, Delaune E, Pilot F, Chopin E, Machuca-Gayet I, Vernay A, Bertrand S, Rual JF, Jurdic P, Hill DE, Vidal M, Schaeffer L, Goillot E: **CKIP-1 regulates mammalian and zebrafish myoblast fusion**. *J Cell Scienc* 2012, **125**:3790-3800.

76. Meriane M, Roux P, Primig M, Fort P, Gauthier-Rouvière C: **Critical activities of Rac1 and Cdc42Hs in skeletal myogenesis: Antagonistic effects of JNK and p38 pathways**. *Mol Biol Cell* 2000, **11**:2513-2528.
