# Supplementary material for: RNAseq analysis of fast skeletal muscle in restriction-fed transgenic coho salmon (Oncorhynchus kisutch): an experimental model uncoupling the growth hormone and nutritional signals regulating growth
Source: BMC Genomics. 2015 Jul 31;16(1):564. doi: 10.1186/s12864-015-1782-z (PMC4521378; doi:10.1186/s12864-015-1782-z)
Supplement: Additional file 11: — Quantitative PCR primer sequences, PCR efficiencies and melting temperatures for genes analysed. (DOCX 143 kb) [file 12864_2015_1782_MOESM11_ESM.docx]

| Gene name | Gene symbol | Forward | Reverse | Product size (bp) | Efficiency  (E%) | Tm  (C^o^) |
| --- | --- | --- | --- | --- | --- | --- |
| *dedicator of cytokinesis protein 1* | *dock1* | AGGCAAGATGGATGATGAGG | AGTTTCAGGGTCACCCACAG | 150 | 94.5 | 87.5 |
| *dedicator of cytokinesis protein 5* | *dock5* | CTACGCCCACTACATCAGCA | CGCAGAAACACACCAATCTG | 149 | 93 | 83.6 |
| *crk-like protein* | *crkl* | ACACGGAGTGTTTTTGGTGAG | GTCCCCTATGCGGAACTGT | 160 | 102 | 86 |
| *cadherin15 (m-cadherin)* | *cad15* | TCCTGAGTGCTGTGGATGAG | TTCCCCTGTCTCCAGCTCTA | 152 | 99.8 | 86.5 |
| *myomaker* | *tmem8c* | AGCCTACATTGCCAAGATGC | TCACACGCATGGTAAATCGT | 147 | 99.5 | 87.5 |
| *integrin beta 3* | *itgb3* | ATGTGATGCCCCTCTACCAG | GAACACCCAGCAACTCCAAT | 150 | 103 | 86.2 |
| *integrin beta 1* | *itgb1* | GGATTACCCCATCGACCTCT | TCAACGAAGGAACCAAAACC | 150 | 100.5 | 83 |
| *growth hormone* | *gh* | TGAGACCAATCGACAAGCAG | TGATGCCCACTTTGTGGTTA | 180 | 97.6 | 84.5 |
| *insulin-like growth factor 1* | *igf1* | ATGTACTGTGCCCCTGTCAAG | CTTGTCTGGGTGCTGTGCT | 150 | 96.5 | 88 |
| *growth hormone receptora.2* | *ghra.2* | AGGAGGAGGAACAGCTCACA | GACATCACTGAACTCCCCAAA | 150 | 99.9 | 86 |
| *growth hormone receptorb.1* | *ghrb.1* | ACTCTGCAGACGTGTCGATG | CACCTCACCCTGACCTCATT | 160 | 94.3 | 87.2 |
| *insulin-like growth factor receptor 1* | *igfr1* | AGCCAAGAGTGACGTGGTCT | GAGATTGCCGTTAGGGTTGA | 136 | 102 | 86 |
| *caveolin 3a* | *cav3a* | AGGCAAACGGACATCATAAA | TGTAACTGGCCTTCCACACC | 117 | 98 | 85.1 |
| *caveolin 3b* | *cav3b* | GCAGAGTCAGGGAGAGTAGT | AGCAGCTGTCCACTGGCATT | 117 | 96 | 87.6 |
| *cell division cycle 42a* | *cdc42a* | GGTGGATTCTCTGCTTCGAG | GGGAGCCCATCACTAAATCA | 152 | 95 | 85.4 |
| *cell division cycle 42b* | *cdc42b* | GAGCCCCATGCTAAGCTCAT | GAGACCCCATAGATGCATCT | 152 | 96.5 | 86.2 |
| *cysteine and histidine-rich domain containing 1a* | *chordc1a* | TATGCAGCCGACAGTGACAT | TAGTTCTGCCTTGCTGCTGA | 202 | 99.7 | 85.3 |
| *vasodilator stimulated phosphoprotein a* | *vaspa* | AAGCCACCTGCTAAGACAGC | AACTCCTTCCGCATCTCATC | 117 | 98.7 | 84.2 |
| *vasodilator stimulated phosphoprotein b* | *vaspb* | GCAAAGAAGGATGAGCCCGG | AGCTCTTTTCTCACTTCCTC | 117 | 96.6 | 86 |
| *trio Rho guanine nucleotide exchange factor* | *trio* | CTCCGCATCAGGAGAAAGTC | ACTCTCCCCCAGGTTACAGG | 198 | 101 | 83.3 |
| *unc-45 homolog B* | *unc45b* | TATCAGACAGGCAGCCACAG | TCATCTTGGTGCACAGCTTC | 200 | 102 | 88.1 |
| *pleckstrin homology domain containing, family O member 1* | *ckip1a* | GGATTACGAGCGTTCTGAGG | CCAGGATGCTGTTCTTGGAT | 200 | 100.5 | 88.2 |
| Rho GTPase activating protein 26 | *graf1* | GGTCTTGCCATGGATCCTAA | GCCTTGATGAAGCTCCTTTG | 152 | 99.2 | 84.9 |
| *60S ribosomal protein l13* | *rpl13* | AAAGAGTACCGCTCCAAGCTC | CTGGCCTTCTCCTTCTTGTG | 149 | 99.8 | 87 |
| *60S ribosomal protein l27* | *rpl27* | CCAGGTCTCCTGTCGATCAT | CATAGATGGGCACTGTGTGG | 140 | 98.2 | 88.5 |
| *elongation factor 1 alpha* | *ef1a* | GCAAGAACGACCCTCCAAT | CGGTCGATCTTCTCCTTGAG | 157 | 96.8 | 89 |
| *beta actin* | *ßactn* | CCAGGTCTCCTGTCGATCAT | CATAGATGGGCACTGTGTGG | 151 | 100.7 | 86 |

Tm: Melting temperature

SEQUENCES USED IN THE PRESENT STUDY FOR PRIMER DESIGN

>Dock1

ATGTCTCGCTGGGTGCCTACGAAGAAGGAGAAGTACGGAGTTGCAATCTACAATTATGATGTCCGTGGAGACGAGGAGCTGTCGCTACAGATTGGAGACACAGTCCACATACTAGAGACATATGAAGGCTGGTACAGAGGTCACAGGCTGAGGAGGAAGTCCAAGAAAGGGATCTTTCCTGCCTGTTACATTCACCTCAAGGAGGCCACCGTTGAAGGCAATGGGCACAAGGAGACGGTGATTCCCAACGAGCTGCCTCTGGTCCAGGAGGTCACCACCACCCTGCGGGAGTGGGCTTCCATATGGAGAGACCTCTACGTGGGGGACAGGCGTGAGATGTTTAACTCTGTCAGGGACATGATCTATGACCTGATCGAGTGGCGATCTCAGATCCTGTCAGGGACGCTCCCACAAGACGAGCTGACTGAGCTTAAACAGAGAGTCACCTCTAAGATCGACTACGGGAACAAGTACCTGGATCTAGACCTCGTGGTGAGAGATAAAGATGGCAACATCTTGGACCCCGACTCTACCAGCACCGTCTCTCTCTTCAGAGCCCATGAAGCTGCTTCCAAACAGATTGAGGACAGGATCCAAGAGGAGAAGTCTCAGAAACAGAATGTAGATCTGAGCAGGCAGGCCAAGTTCGCCCAGACACCGTCCTTCGCTCTCTTCGTCACCCTGAAGAACGTGGTGTGTAAGATCGGAGAAGATGCAGAGGTCCTCATGTCGCTCTACGACCCTGTTGAGTCAAAGTTCATCAGTGAGAATTACCTGGTGCGTTGGTCCAGTCAAGGCCTGGTGAAAGATATAGACCAGCTCCATAACCTGAGATCAGTCTTCACTGACCTTGGCAGTGAGGATTTAAAGAGAGAGAAGATCAGCTTTGTTTGCCAGATCGTTAGAGTCGGACGGATGGAGCTACGTGACAACAACACCAAGAAGCTGACCTCTGGGCTGAGGAGGCCCTTTGGAGTGGCAGTAATGGATGTGACTGACATCATAACAGGCAAGATGGATGATGAGGACAAGCAGTATTTCATCCCCTTCCAGCCGGTGGCGGGGGAGAGCGACTTTCTCCAGACCGTCATCAACAAGGTCATCGCCGCCAAGGAGGTCAACCACAAGGGACAAGGTCTGTGGGTGACCCTGAAACTGCTTCCGGGTGATATCCATCAGATCAGGAAGGACTTCCCTCACCTGGTTGACCGTTCCACAGCTGTGGCCCGCAAGATGGGCTTCCCAGAGATCATCATGCCAGGTGACGTGCGTAACGATATCTACGTAACCCTGGTTCAGGGAGAGTTTGATAAAGGCAGCAAGTCCACCCCCAAGAATGTCGAGGTCACCATGACAGTCTACGACGAGGACGGAAAGAAACTGGAGAACGTGATGTTCCCTGGTGCTGGAGATGAGGGCATCTTGGAGTACAAGTCTGTCATCTACTACCAGATCAAACAGCCACGCTGGTTTGAAACCATCAAGGTTGCCATCCCTATTGAAGACGTGAACCGAAGTCACCTGCGATTCACCTTCCGTCACCGATCATCTCAAGAATCCAAAGACAAATCGGAGAAGATATTCGCCCTGTCGTTTGTGAAGCTGATGAGGTACGATGGGACGACCCTGAGGGACGGAGAGCACGATCTCATCGTATACAAGGCGGAGGCTAAGAAGCTGGAGGACTCGTCTCTATATCTGAACCTGCCTGCTACTAAGGTGGAGCTGGAGGAGAAGGGTTTGTCCACTACAGGGAAGGGTACACACAACCTGGGCAACTGTACCATCAGCAAGGACTCCTTCCAGATAGCAACCCTGGTGTGTTCCACCAAGCTCACACAGAACGTGGATCTGTTGGGCCTGCTGAAGTGGCGCTCCAACACCAGCCTCCTACAGCAGAACCTCCGACAGCTGATGAAGGTCGAGGGAGGAGAGGTCGTCAAGTTTCTGCAGGACACCCTTGATGCCCTGTTTAACATTATGATGGAGAACTCAGACAGTGACACGTTTGACACCCTGGTGTTTGATTCCTTGGTATTCATAATTGGACTCATTGCAGACAGAAAGTTTCAGCACTTTCACCCCGTCCTTGAGACCTACATTAGGAAGCATTTCAGTGCTACTCTGGCGTACACGAAGCTGACCAAGGTTCTGAAGAACTATGTGGAGAATGCAGAGAAGCTGACAGAACAGCTGCTGAAGGCCATGAAAGCTCTGGAGTACATATTCAAGTTTATAGTGCGTTCCAGGGTCCTCTTCAACCAACTGTATGAGAACAAAGGAGAAGCAGACTTCATGGAGTCCTTGAGGAATCTCTTCACTTCCTTCAATGACATGATGAACATCAACTCTGAGAACACAGGCATGGTAAAGGGTGCAGCGTTGAGATACGTCCCCACCATCGTGAACGACGTCAAACTGGTCTTCGATCCCAAGGAGCTCAGTAAACTGTTCACAGAGTTCATTCTGAAGGTTCCTCTGGGTCGTCTGGTCAAACAGAAGCTAAACTGCCTCATCGACATCGTCCACAGTGATCTCTTCACACAGCATGACTGCCGGGAGATCCTGCTTCCTCTGATGACAGAACAGCTGAAGTTTCATCTAGAGAACCAGGAGGAACTGGAGGCCTGCTGTCAGCTGCTGAGTAACATACTAGAGGTCCTCTACAGGAAGGATGTGGGTCCTACTCAGTGGCATGTCCAGATCATCATGGAGAAGCTCCTGAGGACCGTCAACAGAACAGTCATCTCCATGGGCAGGGACTCTCCTCTCATTGGCAGCTTTGTGGCCAGCATGACCGCTACTCTGAGACAGATGGATGACTACCACTACACTCACCTGATCAACACCTTCGGCAAGATGAGGACCGACGTTGTGGATTTCCTGATGGAAACATTCATCATGTTCAAGGACCTCATTGGGAAGAATGTCTACCCTGGCGACTGGGTCATCATGAACATGATGCAAAATAAAGTGTTCCTGAGGGCCATTAATCAGTATGCAGCCGTGTTAAACAAGAAGTTCCTGGATCAGACCAACTTTGAGCTTCAGCTGTGGAACAACTACTTCCACCTCGCCGTGGCGTATCTTACCCAGGAGTCCTTGCAGCTTGAGAACTTCTCCAGCGACAAACGATCCAAGATCTTCCAGAAGTACCAGGACATGAGGAGACAGATCGGCTTTGAGATCAGAGACATGTGGTATAATCTAGGTCCACACAAGATCAAGTTCATCCCAGAGATGGTGGGTCCCATCCTAGAGATGACTCTGGTCCCAGAGATAGAGCTGAGGAAAGCTACCATCCCCATCTTCTTCGACATGATGCAGTGTGAACACA

>dock5

TTTCTACAGGACACACTTGATGCCCTGTTCAACATCATGATGGAGACTTCAGAGAAGGACACCTATGACAATCTGGTCTTTAATGCTCTGGTGTTCATAATCACACTGATTGGAGACATCAAGTTCCAGCACTTTAACCCAGTACTGGAGACATACATCAACAAACACTTCAGTGCCACTTTGGCTTATATGAAGCTGACCAAGGTTCTGAATTACTATGTGGGCCATGCAGATAAGCCTGTCCTAACAGAGGGGCTGTACTCAGCCCTCAAAGCCCTCAAGTACCTGTTCAGGTTCATCGTGCAGTCCCGGGTCCTCTACCTCAGATTCTATGGGACCAGTGAGGATGCTTTCTTCAACTCCATACGGACACTCTTCCTGTCCTTCAACACACTCATGGACAGACCGCTGGATGAGGGAGTGAAGATAAAGGGGGCGATACTAAAATACCTTCCCACCATCATTAATGACATCAAGAATGTCTTTGATCCTGTGGAGCTCAGTGTTCTTTTGACTAAGTTCATTGAGAGCATCCCTGACTCTCAGCTGGTGCGCCAGAAACTTGGTTGCATGTGTAAGATGGTGGAGAGTGACCTTTTCAAACAGCCAGAGTGTCGAGATGTCCTCTTGCCGCTGGTGACAGACCAGCTGAGTGGGCAGCTGGATGACCACTCCAATAAACCAGACCACGAGGCTTGTGTTCAGCTGCTCAGCACTGTGCTGGACAACCTGGACCGCAAGAATGTGGGTCCAACCCGGGGCCATGTCCAGCTGATAATGGAGCGGCTGCTTCGCAGGGTCAATCGCACTGTCATAAGCATGAGCAGAACCTCCACTCTCATTGGTCATTACCTAGCCTGCATGACCGCCATCTTGAAGCAGATGGATGACATGCACTACGCCCACTACATCAGCACCTTCAAGACCAGACAAGACATCATTGACTTCCTGATGGAGACATTCATCATGTTTAAGGACCTGATGGGGAACGTTTTCCCCTCTGACTGGATGACCATGAACCTCCTGCAGATTGGTGTGTTTCTGCGGGCCATCAACCAGTACTCTGAGGTCCTCAACATGTACTTCATGGACCAGACCCACTTTGAGCTGCAGCTCTGGAACAACTACTTCCATTTGACTGTTGCATTCCTTACCCACAAGTCATTGCAACTGGAATCCTTCTCTCAAGAAAAACGGAATAAAATACTGAACAAGTACGGAGACATGAGGAAGAGCATTGGCTTTAAGATCCGAGATATGTGGTATAATCTTGGCCCCCACAAGATGAAGTTCATCCCGGCCATGGTGGGGCCCATCCTAAAGGCTACCCTGGTGCCTGAGCCAGAGCTGAGGAAAGCCACCATCCCCATCTTCTTTGACATGATGCAGTGTGAGCACAACTTCACTCCCAGCCGCACCTTTAACATGTTTGAGAATGAACTGATCACCAAGTTGGATCAGGAGGTAGAGGGAGGCCGTGGGGATGAACAGTACAAAATCCTGCTGGAGAAAACACTACTGGAGCACTGCCGGAGGCACAGATACCTGTCTCAGTCAGGGGAGGAGCTGGCTCTGCTGCTCAGCAGTCTGCTGGAGAAGCTACTGGCCTACCGCACCATCACACATGACGAGAGCCCTGAGCTCCGCATGAGCTGCACCGTTAACGTCCTGAACTTCTACAAGGAGATAGAGAGAGAGGAGATGTACATCAGGTACCTGTACAAGCTGTGTGACCTCCACAAGGAGTGTGACAACTACACTGAAGCTGCCTACACACTGCTGCTGCACGCTAAGCTGCTGAAGTGGTCTGAGGAGCCGTGTGCAGCTCACCTGACCCAGAGAGACGGCTACCAGGCCTCCACACAAGGACAACTCAAGGACCAGCTCTACCAGGAGATCATCAACTTCTTCGACAAGGGCAAGATGTGGGAGGAAGCCATCGTACTAGGAAAGGAGCTGGCAGACCAGTATGAGAATGAGATGTTTGACTTTGAGCAGCTCAGTGCCTCCTTGAGGAAGCAGGCTCAGTTCTACGAGAACATTGTGAAGGTGATCCGGCCCAAGCCAGACTACTTCGCTGTGGGATACTACGGAGTGGGCTTCCCTTCCTTCCTCAGGAACAAGATGTTCATCTATAGGGGGAAGGAGTACGAGAGGAGAGAGGACTTTGAGGCCAGACTTCTCACCCAGTTCCCCAACGCTGACAAGATGAAGACGACCACGCCGCCCGGCGAGGACATCAAGACCTCTTCCTCACAGTGCATCCAATGTTTCACCGTCAAGCCTGTTCTAGAACTCCCTTCAAAGTTCCAGAACAAACCCGTCTCTGAGCAGATCGTCAGCTTTTACATGATAAATGAAGTGTATCAGTTTCAGTACTCCAGACCGGTGAGGAAGGGAGAGAAGGACCCAGACAACGAGTTCTCGAACATGTGGATCGAGCGAACCACTTACACCACGGCCTACAAACTGCCTGGGATCCTGAGATGGTTCCACGTCAGGTCTGTCTCCACGGAGGAGATCAGCCCATTGGAGAATGCCATGGAGACGATGCAACTGACCAATGAGAAGATCAGCAACATGGTGCAGCGTCACCTGAACGACTCCAACCTTCCCATCAACCCCCTCTCCATGCTGCTCAACGGCATCGTGGACCCCGCTGTCATGGGGGGCTTCGCCAAGTACGAAAAGGCCTTCTTCACTGAGGAGTACAACCTGCAACACCCAGAGGACCGAGATAAGCTGTATCGCCTCAAAGACCTCATCGCATGGCAGATCCCGTTGCTGGGGGGAGGGATCTCTCTACATGGTAAGAGAGTGACAGACGACCTACGACCTTTCCACGAGCGCATGGAGGAGTGCTTCAAACACATGAGGAAGAAGGTGGAGAAGGAGTACGGCGTGAGGGACCTGCCGGATATGGATGAGAGGAAGTCAACTCGTCCGCGCTCTGTCCTTCGCTCCTTCCGTCAGTCTGTCATCTCCATCTGCTCCCTCCAG

>CRKL

CATGGCCGGAAATTTTGACGCAGAGGACCGTGCAAGTTGGTACTGGGGTAGATTAAGTAGACAGGAGGCAGTTTCACTTTTACAAGGACAGAGACACGGAGTGTTTTTGGTGAGAGACTCAATTACAAGTCCAGGCGACTACGTGCTGTCAGTTTCAGAGAATTCCAAAGTCTCGCATTACATAATCAACAGCATCAGCAACAACCGGCAGTCTGGCGCAGGCCAGGCGCCTCCACAGTTCCGCATAGGGGACCAGGAGTTTGACGCCCTCCACTCGCTGCTGGAGTTCTACAAGATCCACTACCTGGACACCACCACTCTGATAGAGCCCATCAACAAGGCCAAACACTCTTTCTTGGTCAGCGCAGGTGGTGGCGGCCCGCCGCAGCGGCTGGAGAACGAGTATGTCCGTGCCGTCTTTGATTTCCCAGGCAACGACGATGAGGACCTTCCTTTCAGAAAGGGCGACATCCTGCGGGTTCTGGAGAAGCCTGAGGAGCAGTGGTGGAATGCTCAGAATTTAGAGGGGCGTGCCGGGATGATCCCTGTGCCCTACGTGGAGAAGTACCGACCGGCCTCTCCCACCTCGGGGGGCCTTGGGACAGGGGGTCCAGGTGGGGTGGGCTCGGTAGACGGCTCAAGTGTTCAGGGCCCTCCTCTGCTAGACCCGAGCCAGTACGCCCAGCCCACACCTCTGCCCAACCTGCAGAACGGACCCGTCTTTGCCAGGGCCATCCAGAAGAGGGTGCCCAATGCCTACGACAAGACCGCCCTTGCCTTAGAGGTGGGCGACATGGTGAAGGTGACAAAGATCAATGTGAACGGCCAGTG

>m-cadherin

GGAGGACTTGAACCCAGCGGCCCTGTATCCATGGAGACAGAGGAGCAGTGGAGGTCTGAGCAGGGTGAAGAGGGACTGGATCATCCCTCCAATCAGGGTGCTGGAGAACAGCAAGCAAGTCCCTGAAAACCTGGTCCAGATCAAATCAGACAAGATTTTCACTGGAGAAGTGATCTACAAGCTGGAAGGACCAGGGGTTGACCAGGACCCTAAGGACCTGTTTGAGATAGACGATAAAACAGGCTGGATCAGGAGCATGATTCCCCTGGACAGAGAGAAAAACAGAAGCTTCACGCTGAAAGCCTTTGCCCTGTCGCCCAGTGGAGAGAGACTGGAGAATCCCTCCACCATTGAGATCGTAGTGCTGGATCAAAATGATAACCGGCCCAACTTCTCCCAGAAAGAGTTTGCTGGATCCGTTTATGAATTCTCCGTACCGGGCACATCTGTGATGTCTGTGACTGCGACTGATGCTGACGACCCAACCACAGACAACGCTATCCTGAGCTACTCCATCATTGGCCAGGAGAGCATCCCGCCTCTCCGCATCAATAAGACCATGTTTGGCATCAACAACGAGACAGGGGCCATTTACACACGAGACGTGGGCCTAGACTGGGAGGTGGTTAAAGGTTTTAGGTTGACACTGCAGGTTGCTGACATGTCAGGCATGGGGTTAATCAGTCTTGCCAATGCAGTCATACATGTGACTGACATCAACAACAATCCCCCACAATTCGCCCCAGACCTGTACACTATGTCAGCTGTGGAGAACAAAGTAGACTTCGTGATTGGCTGGGTCAACGCCACAGACAATGATGAGCCAGGGACAGGAAACTGGGAGACCAAATACACTATCGCCAAGGGCAACCCCTCCAGGAACTTTGCCATTCGCACAGACCCTGTGACCAACGAGGGTATTCTGTCAGTGGTGAAGGCTCTGGACTACGAGACCCAGGAGGTGTATACTTTGACTCTGACAGTGGAGAATGTGAACCCTCTCAGCATCAAGGCCCCCAAGGACCCCGTAAGCAGTGCCACAGTGGTGGTGACCGTGGTGAATGAGAACGAGGCCCCACGCTTCAATAAAGACCCCATAGAGATCGTGGTTCTTGAGTCTGTGGACCCTGGCACCTTGCTGGCCAGTAACATCGCCTACGATCCCGATAATGCAAAACTCAAGTATGACATACTCAGAGATCCTGAGGGATGGCTTATAATCAATCACGAGACTGGAGAAATCGCAGCCAGGAAACCGTTCAATGTCCTCTCCCCTCACGTCAAGAACAACATTTACAATGCAGTCATCAGAGTCACAGACACAGACGCTGGTGGAATCTCGACCACAGCGTCTCTGGTGATCACGTTGTGGGAGACCAATGACTTCCTCCCTCAGCTGTTCCCCCTGATGGGGACGGTGTGCAGCGAGTCAGGCCGGGAGACATCTGGTCTGTTCCTGAGTGCTGTGGATGAGGACCTGCCCCCTCACGCTGAACCCTTCACCTTCCACCTGCAAGACATGAATGTGTCTGCCAACTGGACCATCATACAGGTCAATGAGACTCATGCAGTGCTGCAACCCCTGGTAGAGCTGGAGACAGGGGAATATGCTGTCACTGTGTTGGTGACTGACTCTGGCACCCCAAGTCTCAGTGCATATGCTCAGGTCAATGTGACTGTGTGTCCGTGTGGGAAAGACGGTGAATGTAAGACCGAAGCTGCTGCCATATTCGGAACACGAGTTGGTGTCAGCTTCATTGCCTTACTGGTCATCATGGCCAGCATCGCACTCCTGCTGTTGTTGCTACTCCTGGCTGTAGCCGTGGGTAACTGCAGACGGCATCATATGAAGAAAGGAGAAGGGCTGTTGGTGGGGGATTCAGACGAAGACATCCGTGACAATATCCTGAACTATGACGAGCAGGGTGGTGGAGAGGAGGATGAGAATGCCTTCAACATTGACCTTCTGAGGAACCCAACGGATGTGGGACCACTCCCAATATCCTACTACCCTCCCGTCTCTGGCATCCCCAGGGGAAAGCAGCCACTCAGGAAAGACTACCCAGACAACCTGCCCTCCCCCTCGTATCCACGGAAACCCCCAGCGGACCCCACTGACATTGAGGACTTCATCAATGATGGCCTGGAGGCAGCAGACCATGACCCCAACGTCCCTCCCTACGACACAGCTCTGATCTATGACTATGAGGGTGATGGCTCCCTGGCAGGCAGCCTGAGCTCCATCGCCTCAGCCAGCTCAGACGGCGACCAGGACTATGACTACCTCAACGACTGGGGACCACGCTTTAAGAAACTGGCCAACATGTACGACCCACGC

>myomaker/tmem8c

CATGGGAGCCTACATTGCCAAGATGCTGCTGCCCACCGTTAGTAGCCTGGTGTTCCTGCCTGCAGCCAGCGTGGCCACCAAGAGAGGCTTCCACATGGAGGCCATGGTCTACTTCTTCACCATGTTCTTCACCACGATTTACCATGCGTGTGATGGGCCAGGCCTCTCCATCATATGTTTCATGAAGTATGAGATCCTGGAGTACTTCAGCGTGTACGGAACAGCCATCTCCATCTGGGTCACACTGATAGCTTTAGGGGATTTTGATGAGCCAAAGCGTTCTACTCTGACCATGTTTGGGGTTTTGACCTGTGCTGTGAGGATCTACCAGGACCGCTGGGGCTATGGGATCTACTCTGGACCAATAGGATCAGCAGTATTCATAATCACTGTCAAATGGTTACAGAAAATGAAAGAGACAAGGGGCCTCTACCCCGAGAAGAGAGTATATACTCAACAGGTGGGACCAGGATGCTGCTTCGGTGCCCTGGCTCTGATGCTCCGCTTCTACTTCGAGGAGTGGGACTACGCCTATGTCCACAGTTTCTACCACCTGTCTATGGCGGTGTCCTTTGTCTTGCTGCTACCCAAAAAGAACCGCTACGCGGGGACCGACGGCAACCCCGCCAAGCTCAGCTGCTACACGCTCCTCTGCTGCACACCATTTCCTGCTTCTACGAAGGAGAAGAAGGACAAGAAGAAGACCCCGTCTCGAACCATTTGGACCATTCCCACTGAGCGGCCGTGGACACGGGCCTGCAACTCACCAACTCTCCCTCTGTACAACCCACCTAGTACACCTGTTAAGAAAGCACTGGA

>itgb3

TCCCACGGTAGGTCACATTGGTGAAAGTGGTGGTGGCTCCTTTGTACAAGGGGTTGTTAGCCGTATCCCACTTGGCCTTAGCCCTCTCCTCCTCAAACTTGGCGAACTCTCGGCGGTCGTGGATGGTGACCAGCAGCTTCCAGATGAGCAGGCCAGCCAGACCCAGAAACAAGATGGCCCCCGCCACCGCCATGAGAACCACCAGGATATCAGGCCCTTTAGGGCAGTCAGGCTCCATGACATACAGGATGGACTTTCCGCTGGCATCCTCGTAGTACTGGAAGCGCTCTACACAGTCATTCTCATCCTTATAGGTGCAGTTCACAGCATTCTTCTCATAGAAGACTAGTTCTTCCACCTTCTGAATTTCATCCCTGCAGATTCGGGCACAGCTTTTCTCCTCAAAGAGACGGCCTCTCTTGAAGTGCTTACATTCAACACATTCCTTCTTGATGCTGCAGGCATCAGGGCAGGTTGGACACCTCTCACAAGTGGCCCCGTAGGCCCCTGGCTGGGTGCACTCACAGGCCCCACAGACACACTGGCCCCGGCCGCTGCACAGCAGGCCCATGCTGGACATGCAGGTGTCTGTCCGGGTTGTGCAGTTACAGTTCTCCCCCATCCAGTCAGAGCCACACTTGCAGAAGCCACAGTCACATTTCCCATGGCCTGAACAGATCTCTCCTTTGAAGCGCAGGCAGTTGAAGTCGTCACATTCGCAGTACTTGCCCCACACCTTTCCAAAGCCACTAGTGTGGCAGGAGCATTGGCCACACACACAGTTGCCCCTCCCACTGCAGATGGGGTCATCAGGTTTGGGGCTGCAGTTGCCCTGGTCTGAGGGCCTGTAGTCCCCGTCGGCACACTCACAGCGGTCTCCCAGGCGCCCCGGGTGACACTGGCACACACCACACTCGTAGGTCCCATTTCCCTGGTTACAGAGAGGGCTCTCGGGCTGGGCTGTGGCCTCACAGTCACACCCGCAGGCGAAGTGGACCGTAACCTCCAGGGCGTCCTTGAAGCCCACGGGTTTAATGGTGAAGGTATGGTTCTTCTCCTTAGGGCAACCGCGTAACCTTGCCTCCACACTGAACGACACTGTGTCTCCGATCTTAAGTCCGGAGCAGGACTTGAGTCCCTGGATGAGCTCTCCGTTGAGGCAGGTGGCGTTGAAGGACAGATTCAGCTCCTCTGGAACACCCAGCAACTCCAATTCCACCTTGGAACGGATCCTCTCGTAAGCATCCAGGATGAGTTGGATCACATTCCCAGAGTCATCCGACAGCGTTCCAACCGTGGTGCCAGGAATCAGCTGGCTGTAGTTCTGGTAGAGGGGCATCACATTGTTGGTCACAGCGAAAATGAGGTTGATGTTGTTCTCAGACATCTTCTCTGTGATCAGAGCCAGAGATGGGTAGTCCAGGATTGACGACTTGTCATAGTTGTTGTCTCTGTCGATGTGACACTGTCCATCATTGGGCTGCACTAGACCAGCCAGCCGTCCGTCCAGGGCTATGTGTGTCGTAGCGTCGGTAGTGAACACCAACAGGTGGGAGGCATCCCCACGCCAGCCTATCTTGTCCTTGCACACCACTGCCTGTATGACCGCGTCAAAGCCTCCCTCTGGAGAGTCTCTGTTACGAGACACCTGCTGCTTCGCCACCTCCTCGGTGAAGCGCTCCACCTGCTTGGTCAGAGACAGCACGTGCTTGTAGCCAAACTGAGGCAGGCACCGCGTCTGGATCCCATAGCAAGGGTTACTGACGGCTTCCTCAGGGTAGGTGTACATATATGGGGACAGGGGCTTGTCCACAAAGGCCCCAAAGCCCATACGCAGGTTGCTGGTGGTGCGGCCCATGGCTGCAGCCAGCTGGTTGCCCAGGGAGCGCAGGCGGGCCAAGTCGTCCCTCATGGAGTAGGAGAGGTCCATCAGATAGTACAGGTCAACTGGGTAGTCCTCCACCTGCTTCACTGTCACTGTGAAACGCCTGGCATCATCTGGTCTCAGAGTGAGGCGGAGTTTCTGGGGTCTGATCTGGGTGACGTCGTCAGCAGCCCCCGATGCCTTGTCACTGAGGGGCGTGTTTTCTTGCACACTGAGGGTACTGAAGGGGAACTCTAGCCCCCCCTTCGTACAACCCCCATCCAACAGGTTCTGCTTCAGGTCACAACGGGACACACTGG

>ITGB1

TATGTTGACTCGGCAATAAATCACGAGGAGAAGATGGACCTGAAACTACCTCTGATATCAACATTGTTAGCAGTCATTTGTTACAGCAGTGCTCAGCAAGAAGGGAACGAGTGCATCAAGGCCAATGCCAAGTCCTGTGGGGAGTGTATTCAAGTGGGAGCGAAATGTGGATGGTGCACAGACCCAGAGTTTCTTAAGCAGGGTGAGGCCACGTCGACCCGCTGTGACGAGCTAGAGTCCCTGCGGAAGAGAGGTTGCAGTGGCGCCAAGGTAGAGAACCCCCGAGGCAGCCAAGAGGCCCTGAAGAACAAGACTGTGACCAACCGCAACAAGGGAGCAAAGAAACTCAGACCAGAGGATATCACACAGATCCAGCCCCAGAAACTCACCCTTAGCCTCAGATCTGGTGAACCCCAGTCTTTTAATCTGAAGTTCAAACGAGCTGAGGATTACCCCATCGACCTCTACTACCTGATGGACCTCTCCTACTCAATGAAGGATGATCTGGAAAATGTAAAGAACCTGGGAACACAGCTGATGCTGGAGATGTCAAAGATCACATCTGATTTCAGAATCGGTTTTGGTTCCTTCGTTGAGAAGACTGTGATGCCTTACATAAGCACCACCCCAGCCAAGCTGCTGAACCCCTGTACTGGGGACCAGAACTGCACCAGTCCATTCAGCTATAAGAACGTCCTGAAGCTGACCAGCAATGGACAGGAGTTCAACACCCTGGTGGGACGACAGCAGATCTCTGGAAACCTGGACTCACCAGAGGGAGGCTTTGATGCCATCATGCAAGTGGCTGTCTGTGGTGAGCACATTGGCTGGAGGAACGTCACACGTCTGCTGGTGTTTTCCACTGATGCTGGCTTCCACTTTGCTGGAGATGGGAAACTGGGAGGGATTGTTCTGCCCAACGATGGGAAATGCCACCTGGAGAACAATATGTACACCATGAGCCACTACTATGACTACCCCTCCATCGCCCATCTGGTTCAGAAACTAAGTGACAACAACATCCAGACAATCTTTGCCATCACAGAGGAGTTCCAGCCTGTTTACAAGGAGTTGAAGAATCTCATTCCTAAGTCTGCAGTTGGGACACTCTCTGCAAACTCCAGCAACGTTATCAACCTCATTATTGATGCCTACAATTCTCTTTCCTCAGAGGTGATTCTAGAGAACAGTAAGCTAGCAGAGGGAGTGACCATTGCGTACCAGTCACGCTGCAAGAACGGAGTGACTGGTGAGGGAGAGATGGGAAGAAAATGCTCCAACATCTCCATCGGAGACGAGGTGTCTTTCACCATAAGCATCACAGCTAAGCAGTGTCCTAAAATGGGCAAACCAGAGACCATCAAGATCAAACCCCTAGGGTTCAATGAGGAGGTGGAGATCACTCTGAACTTCATCTGCGAGTGTGACTGTCACAAAGATGGCATTGAGAACAGTGACATCTGCCACAAAGGCAACGGGACATATGAATGTGGAACCTGCAGG

>GH

AAATGGGACAAGTGTTTCTGCTGATGCCAGTCTTACTGTTCAGTTGTTTTCTGGGTCAAGGAGAAGTGATGGAAAACCAACAGCTCTTCAACATTGCAGTCAACAGGGTGCAACAGTTCCACCTACTGGCTCAGAAAATGTCCAACGACTTTGTTCACACAAACCTAGCGGCAAAAAATTCTCTCTCCCGTCTGTGTGATTTTGTGCAGGAAGGCACCCTGTTTTCTGATGAACGCAGACAGCTGAACAAGATATTCCAGCTGGACATCTATAACTCTGACTTCATCATGAGACCAATCGACAAGCAGGAGACTCAGAAGACTTCAGTCCTGAAGCTTCTCCATATCTCTTTCCACCTGATTGAATCCTGGGAGTACTCTAGCCAGACCCTGACCATCACAAACAGCCTAATGGTCAGAAACTCCAACCAGATCTCGGAGAAGCTCAGTAACCACAAAGTGGGCATCAACCTGCTCATCAAGGTTGAGACCTACCTGACCGTCGCTAAGTGCAGGAAGTCGCTGGAGGCCAACTGCACTCTGTA

>igf1

AAACCAACGGGGTATGGCCCCAGTTCACGGCGGTCACATAACCGTGGTATTGTGGACGAGTGCTGCTTCCAGAGTTGCGAGCTGAGGCGGCTCGAAATGTACTGTGCCCCTGTCAAGTCTGGCAAGGCAGCTCGCTCTGTGCGCGCACAGCGCCACACAGACATGCCAAGAACACCTAAGGTTAGTACTGCAGTGCAAAACGTGGACCGAGGCACAGAGCGTAGGACAGCACAGCACCCAGACAAGACAAAACCCAAGAAGAAACCTTTATCTGGGAATAGTCACACATCTTGCAAGGAGGTACATCAGAAGAACTCAAGTCGAGGAAACACAGGGGGAAGGAACTACCGAATG

>GHRa.2

GCTGTGAGATCAGCCTCACTGATGGACCCTGGCTCTATGACGTCATCAGATCCCTCCGTCCAGGCTCCTCATTTGACTGGGTGTAAGTCCAGGGAACAGGAGACGTTTAGGTGTTGGTGGAGTCCCGGAAGCTTCCAGAATCTCACGGAACCCGGAGCACTGCAAATCCAGTACTGGAAGAAGAATGACTTGACGAAGGAATGGAAGGAGTGCCCTGACTACTCCTCCTCTGTGAAGAACGAGTGTTTCTTCAATAAGAACAACACGGTTATCTGGATCAAATACTGTGTGCGGCTCCACTCTGAAAGCCAGAACAAAACCTACGATACCTTGTGCTTCGAATTGCAGGACATTGTGCACCCTGACCCGCCAGTGGCTCTGAATTGGACGCTGCTGAACATCAGCCGGTCGGGCCTGAACTATGACATCATGGCGAGCTGGGAACCCCCGCCCTCGGCCGACGTATCCGTTGGATGGTTGACACTGGTGTATGAAGTCCAGTACAGGAGGAGGAACAGCTCACACTGGAAAGTGTTGGAGCATGAGTTTGGCACCCAGCAGTCTATCTACGGCCTTCAGACAGGAGAGGCGTACGAGGTGCGTGTGCACTGTGCAATGAGAGCCTTCAATCACTTTGGGGAGTTCAGTGATGTCATCTTTGTCCACGTGCCAGAGATTCCCAATAAAGAGTCAACGTTCCCTGTGACTCTGGTCCTGATCTTCGGGGCTGTGGGCGTGGCCATTCTCCTCATGCTCATCATCTTCTCTCAGCAGCAGAGATTGATGGTGATTCTGCTGCCTCCTGTCCCTGCACCCAAAATCAAAGGGATAGATCCAGCATTGCTTAAGAACGGAAAGCTGGACGAGCTGAACTTCATCCTGAGTGGAGCTGGTATGGGGGCTCTCCACTCCTACCCCCCGGACCTGTACCAGGACGTGCCCTGGGTGGAGTTCATCGAGCTG

>GHRb.2

TCATGGCAATCTCCCACATTCTCTTCATCTGCCTCGTCCTCATCCTTCCAGTTTTATCGCAGGAACCCCCCACCTCTAAACAAGCCCTCTTCCAGATCCGCCCCCAGATCACTGGCTGTGTCTCCCATGACATGAACACATTCCGCTGCAGATGGAATGTTGGAGTTTTCCAGAACCTCACAGAACCCAGAGACCTGCGGATATTCTACTACATTAATGATAGGAACATATCTCCCAAAGAGTGGGGTGAATGTCCTCGCTACGCTGACAGGACAAACGAGTGCTTCTTCAATGAAAGCTACACGAAGGTCTGGATGACCTACAGTGTCCAGCTCCGCTCTGGCGATCAGGACATTCTCTATGACGAGGTCATCTTCACTGTGGAAGACATCGTGGAACCAGACCCTCCTATAGCGCTGAACTGGACCCTGCTGAATGTGGGTCTAACCGGGAGCCACTTTGATATCATGTTGAGCTGGGAACCGCCACACTCTGCAGACGTGTCGATGGGCTGGATGACGCTGCAGTACGAGGTGCAGTATCGCGAGGTCAACTCAACACTGTGGAGGACGGTGGACCTTGAAAAGGGAATGCAGCGATCGCTATACGGGCTGCGCTCCAACACAGATAATGAGGTCAGGGTGAGGTGTAAGACGCTAGCATCCCGTAACTTTGGGGAATTCAGCGACTCCATATTCATACACATCCCTACTAAAGAGTCGAGACTTCCAGTTACTGTCCTGCTTGTCTTTGCTGCTTTGGGTTTGGCAGTCATCCTAATGCTGGTAATCTATTCCCAGCAACAGAAGTTGATGGTGATTCTCCTGCCTCCAATTCCTGGTCCCAAAATCAAAGGCATAGACCCAGAGCTTTTTAAGAAAGGTAAGTTGGCTGAGCTGACCTCCATCCTGGGCGGCATCCCTGACTTGAGGCCAGACCTGTACAGCGACGACCCCTGGGTGGAGTTCATCGAGCTGGACATGGAGGAGCCCAACGACAGGCTGACTGTGCTGGACACCCAGTGCTTGATGGACCACTGTGCCTCCTCAGACTGTCCCCCTATCACCATTGGCTTCAGGGATGATGACTCGGGCCGGGCCAGCTGCTGTGACCCTGATCTGCATGACCCAGAGGCCCCCTTCCACTCCCTCCTCCCCAACACCAGCCACGCCCTGGAGCCCTCTTGCCTGGCCAGCACTAAGGCCAGCTCCCCAGTCCAGACCCCCACCACTGAGGATTCTCCCTGGGCCGCACCTGGCAGGGAGGACCTCTACACCCAGGTGAATGAGGTGAGACCAACTGGTGAAGTGCTGCTGACAAATGAGGAGCAGAGGAATGTGGAGGAGAATTCAGAGAAGGATGAGAAGGAAAATGAGAAAGAGAAGAAGAAGAAAGAGTTTCAGCTGCTGGTGGTGAATGCTGATGTGGGAGGCTATACCTCAGAGTTAGATGCTGGGAAGATGAGTGCCAGACTCCCCACTGGGAGAGCCAGCCAGCCTGCTCCAACAGAGGACAGTAGTCTTGTGCAGGGACAGCCCTTCGGAGAGTACCAGAGCCTGTACTTTGAGGCTGAAATGCCCCCCATTCCACCTGCCTCTCCTGTCTCTCCACTGCCCCCTGTCTCTGCCTACACCATGGTGGAGGGAGTAGGCAGGCAGAACAGCCTCCTTCTGAAGCCTGGACCCACACCTGCACCCCAGCCAGTTCTAACCAAGCTTCCCCTGCCCACACCTACACCAGAGGGGTACCTGACCCCTGACCTACTGGGCAACATTACACCAT

>igf1Ra

GGACGTGGACCTTCCCCAGGAGAAGAATTCTGACCCAGGGGTTCTACTGTCGCCTCTTAAGCCCTGGACCCAGTACGCCATCTTTGTCAAGGCCATCACCCTGGTGGTGGAGGACAAACACATCCCGGGAGCCAAGAGTGACGTGGTCTATATACGCACCAGCCCTTCAGAGCCGTCTATGCCAATGGATACCCGGGCGTATTCCAACTCGTCCTCCAAGCTGGTGGTGAAATGGTCACCTCCCCTCAACCCTAACGGCAATCTCACTTTTTACCTGATCCGCTGGCAGCAGCAAGCTGAAGACAAGGAGCTGTACCAACACAACTACTGCTCCAAAGAGCTGAAGATCCCCATTAGAATCTCAGCCACGGGGTTGGCAGACATGGAGGACGACACTAAGCCCACCAAGCCTGACCTGTCTGGGGGGGACAAGGGCCCCTGCTGCCCCTGCCCCAAGACCAAGGAGGACCTGAAAGCAGAGATAGATGACGCATCCTACCGCAAAGTCTTTGAGAACTTCCTGCACAACTCCATCTTCACCCCCAGGCCCCCAGACCGGCGGCGTAGGGATCTATTTGGGGTGGCTAACAGCACCTTGGCGCGGGGTGGCAACACCACAGGCCTGGAGGGTAATGGGACAGACGGAGAATCCCCCGAGAGGGAGTTCCCCTTCATGGAGGACCGGAGCAAGACAGAGTTTATAGAGATCCCCAACCTACAGCCCTTCACTGTTTACCGCATCGACATCCACGCCTGTAACCAGGAGGTCCGTCGCTGCAGCGCTGGAGCCTTCGTCTTCTCCAGGACCAAACCTGCAGACAAAGCGGATGACATGCCAGGCACGGTGAATCAGGAGAGGGATGAGAAGGTGGAGGGGTCGGTGCTGCTGAGGTGGCCGGAGCCTGTCAACCCCAACGGTCTCATCCTAATGTATGAGATTAAGTTTCGCCAAGGCACCGAGCCTGAGAAACATGAGTGTGTGTCACGCCAGCACTACAGAGTGCACAAAGGAGCTCGTCTGACCAACCTAGGCTCTGGGAACTACTCTGCCCGCGTGCGCGCCACCTCCCTGGCAGGAAATGGGTCCTGGACTGAGCCAGTGTCCTTCTACGTAACCCCACGCACACGGGACTACGACATCACGTTCTATCTGGTCATCATCATCCCCATCATAGTGATAATTCTTATCGCCAGCCTCATTACTGCACTCTTTTTTATCAACAAAAAAAGGAACAGTGACAGGCTGGGGAATGGTGTCCTCTATGCTTCTGTCAACCCTGAGTACTTCAGTGCTGCTGAGATGTATGTTCCAGATGAGTGGGAGGTGGCCAGGGAAAAGATCACCATGCACAAGGAGTTGGGCCAGGGTTCCTTTGGCATGGTGTACGAGGGCCTCGCTAAGGGCGTGGTTAAAGACGACCCAGAGACGCGCGTGGCCATCAAGACGGTCAACGAGTCAGCCAGCATGAGGGAGAGGATAGAGTTCCTCAATGAGGCTTCCGTCATGAAGGAGTTCAACTGTCACCACGTGGTGCGTCTGCTGGGCGTGGTCTCTCAGGGACAGCCCACCTTGGTGATAATGGAGCTGATGACCCGTGGAGACCTGAAGAGTCACCTGCGCTCGCTCCGTAAAGAGAACACGTCCAGCCAGGTTCTGCCCCCGCTGAAGAAGATGATCCAGATGGCAGGGGAGATTGCGGACGGCATGGCATACCTCAACGCCAACAAGTTCGTCCACAGAGACCTGGCTGCCAGGAACTGCATGGTGGCGGAGGACTTCACCGTCAAGATCGGAGACTTTGGCATGACGCGAGACATCTACGAGACGGATTACTATCGGAAAGGAGGGAAGGGCCTTCTGCCAGTCCGCTGGATGTCCCCAGAGTCGCTGAAAGACGGAGTGTTCACCACAAACTCTGATGTCTGGTCATTTGGGGTGGTGCTGTGGGAGATTTCTACCTTGTCTGAGCAGCCGTATCAGGGCATGTCCAACGAGCAGGTGCTGCGTTTCGTCATGGAGGGAGGACTCCTGGACAAGCCTGACAACTGTCCTGACATGCTGTTTGAGCTGATGCGGATGTGCTGGCAGTACAACCCCAAGATGCGTCCTTCCTTCCTGGAGATCATCAACAGCCTCAAGGAGGAGCTGGAGCCTCCGTTCAGTGAGATGAGCTTCTTCTTCAGCGAGGAGAACAAGCCCCCAGACACTGAGGAGCTGGACATGGAGGTGGAGAACATGGAGAACGTGCCACTGGACCCTGCACCCAGCAGACCCCCCACTGCTGTTCCTCTACCCTCCCAGGGACCCATGGGGGCCACAGGGGGCTCAGCACCCCCTCCTCCCCAGCAGTTATCCCCAATGCAATGCCCTTCTAATACCCTGAAGGGACCTGGCTCTCCCTCCACCTCAGTCCCGGCTGCCTCTGTCTCGGCCTCCCCAGGCCTGGCCTTGGACAAGCACTCAGGACAGATATCGGCCAACGGGCCAGTGGTGGTGCTGCGGCCCAACTTTGAGGACACACAGAACTACGCTCACATGAACGGGGGGCGCAAGAATGAGCGGGCGC

>comp128137_c0_seq10_rframe1_ORF_cdc42a

AAGACCGTACCCTTGGCCATCACCATGAACCTGGGGAAGATCCCTGGGCTGAAGGGCCTGGTGGCCGGCTCTCAGGGGAAACGTCGTTTCAAAGGTGACCTCACCCTGGACATGATCAGTCCGCCGCTGGGGGACTTCCAACATACCATGCACGTGGGCCGCGGAGGGGACGTGTTCGGGGACACCTCGTTCCTCAGCAACCATGGGGGGGCCACCAACAGAAACGGGGATGTTGATTCTGTCACAAGCCCTGACAACAAGATTGGGGCGTTCTTCTCTCGGACCCTCCGGCGTGTTCGGAAGACACCAGAGCGTCCCAGGGGAGGATCCAAGGACCTGTCCCCGCCACCCCCTCCCATATCCCCCATTATCAAGAATGCAGTGTCCCTCCCCCGACTGGATGTGGACTCACCAAATGGCTGCCCTGCTAAAAACCTCTTCCCCGCCTCTCCTACCTCCCTGGAGGAGACCACTTATGGTTATGGTTTGGAGTCAGGTTTCGCCACTCTGCCCCGCCTCTCCCGTTCAGCAGAGCGTCAGTCGCAACAGGGAGGATCTATTTCCTGTACCCCCAATGCCCATGGCTGCTCGCTCACCGATGTCTCCGCCCTCCTGAAGTCCTCCGCATACCCCACCCTGACTTCTGACCCCGGTCACATGACCCAGTATGAGTCCCTGACCTCCATGGCCTCCTTCACCTTTGACCTGGGGCCCTCCCTCATGAGCGAGGTGTTCGGTTTGATTGACAGCCCTAACGGCCACCTAGAGCCCAGCCATGCCTGGGTGGCAGAGGAGCCAAGCTGCTCTGCATTTGGGTTTGTGACCAATGAAGGCTCGGAGATGGACTCGGAGATGGATGCCACCACTGCCTCATTGGTGGATTCTCTGCTTCGAGAAGACTGTAGCAGCAGGAAGAGTCCATATGGGATGGAGTGGGAAGAGGAGGAAGAGGAGGCTAGGAGAATGGAGATGAATGGAGGTGGGCAGCATCTTAAAGGGGCGGTGCCTGATTTAGTGATGGGCTCCCCGTCTAGACAGAGGCCTGCTATGGAGAGTGAGAGGTTCCAGGGTGCCACAGATGTGCTGGGGGTGCTCTACGGAGTTGGAGGGACCCTGAAGGGGCAGCAGAGGGTGGATCTGGAGGGAGAGGTGACCATGGGCCAGACCATGAAGAAAACACCTTACATCTGCATTACCCCTGATGAGGAGGAGGAAATCAAAGTC

>comp130255_c0_seq5_cdc42b

TTTCTTACTGGATTTTTTATCCCCCTGATTAATTATATTTTAAGGGTAGTCATCTCTGCTACCATGAGTCTGGGAAAACTGCCAGGGATTAAAGGCCTAGGGTCGGGCTCTCAGGGGAAGAGGCGCTTTAAGAGCGAACTCTCCGTGGACATGATCAGCCCACCGCTCCCAGACTTTCGCCACACCATGCACGTGGGCCGCGGTGGCGATGTGTTTGGAGACACCTCCTTCCTTAGCAATCATGGGGGCAAAGGGGAACCAGTGAGCCCCGATTCACCCACCAGTAGCACGAAGACCACCCGCTTCTTCTCCCGCACCCTGAGGCATGTACGCAGGTCCCCTGTGCCCCGTGCCAGGATCGGTTCCCGTGACCTTTCACCCCCACCGCCTCCTGTCTCACCCATCATCAAGAACGCCATCTCACTACCCCAGCTGAACATTGACATGCCCAACGGCTACCAGAGGGTGTTGTTCCCCAGCTCAGAGAGCTCCCCAGATGCCTCCCTCTACAGCTACGGTCTGCAGTCTGGTTTCGTCACACTACCCCGCCACTCCCGCCTTGATAAACAGCTGCAGGACGGCACCGGACTGTTCGCTCAGGACTTTCGCTGTGGCTCGCTCCCAGACAACGGCTTCGCTCTAACGCGCTCCGACTCCTTCACCTCGTTCACAGTGGACCTTGGCCCCTCCCTCATGAGTGAGGTTCTGGGCATGATTGACAGCCCCAGTTGCCTCACTATGCCCAATCACAGCTGGGAGTTGGGGGAAGAGGAAGAGGAGGAGCAGAGCTCTGTGTTTGAGCTGGCTGTGCAGAGCCCCATGCTAAGCTCATCAAACCCTTCCATGTCAAGCACTCCACTCAAAGTGAATGTGAACAACAGGGGAGAGGAGGAGGATGGGAGGTCTCTTGAGACACCAGATGCATCTATGGGGTCTCCTACACAGGTAGAACCAGTTATGGAAGCTGAGAGGTTCCAGAGAGCAGCTGACATGCTGGCGCGTCACTACGGGGGAGGCTCCTTCTCCAGGAGTCATCGCAGCGATTCTGCCTCCTCCTCCTCACCTCTCAGCCAGCCTAAAGTCCCCTACGCCTTCCCTGAGGAAGAAGAGATCAAAGTC

>comp129980_c2_seq12_cav3a

TGGTCAAAGAGGCAGTGTGGGGAGTGGGACAGGGAGTGGCTGTACCCAGGCTTCTGGATTTCCCCTCACTGTGATGTCACTCCTGGAATGCCAGCAGCACCCAGCAGGCAGCTGCCCCTAGTTGTCCTGTCAAACTGTAACCACTGTAGCCATAGCATAGCACAAACACACAAACACACAACACATAGCCTACAGTTACACACATACTTTGTTGACTACAACAAATTCTCGTTAATAATAGCTTATAAACATCTTGTAAACACACTAAAGAATGAACACACACAAACACACACACACATTATTTTACTATCCTTGTGGGGA

>comp130016_c1_seq7_cav3b

TCCCACGACCCCACCATGGCCGACCAGTACCAGTACAACACCAACGAGGAGAAGATTGTGAAGGACAGCCACACCAAGGAGATCGATCTGATCAACAGAGACCCCAAATTGATAAATGAGGATGTGATCAAGGTGGAGTTTGAGGATGTGATCGCAGAGCCCGACGGCACACACAGTCTGGATGGGGTGTGGAAGCTCAGCTACACCACCTTCACCGTGTCCAAGTACTGGTGCTATCGCATCCTCTCAGCCATCTTCGGCATCCCTATGGCTCTGCTCTGGGGCTTCCTCTTCGCCTGCATCTCATTCTGTCACATCTGGGCTGTGGTACCCTGTATCAAGAGCTGCCTGATCGAGTCCCAGTGCATCAGTCGCATCTACTCCCTCTGCATCCAGACCTTCTGTGACCCCTTCTTTGAAGCCCTGGGCAAGATCTTCAGCAGTGTGAAAGTGGCCCTGCGCAAAGAGGTC

>comp127828_c1_seq5_graf1

ATGGGACTGCCTGCTCTGGAGTTTAGTGACTGCTATCTGGACAGTCCCCAGTTCCGAGACCGATTGAAGTCTCACGAACTGGAACTGGACAAGACCAATAAGTTCATCAAAGAGCTGATCAAAGATGGGAAGGCCCTCATCCAAGCACTGAAAAATCTGTCCACAGCCAAGAGGAAGTTTGCGGAGTCTCTCAATGAGTTTAAGTTCCAGTGCATAGGTGACGCAGAGACGGACGACGAGATATGTATCGCAAAGTCTCTACAGGAGTTTGCTGGAGTTTTGCAGAACCTGGAGGATGAGAGAACACGGATGATTGAGAATGCCGATGATGTGCTGATCATGCCACTGGAGCGGTTCAGGAAGGAGCAGATCAGCGCTGCCAAGGAAGCCAAGAAGAAATACGACAAAGAGACAGAGAAGTACTGTGCTGTACTGGAGAAGCACCTAAGCCTATCAGCAAAGAAGAAAGAAGCACATCTCCATGAAGCAGACAGTCAGGTGGACCACGTGCGGCAGCATTTCTATGAGGTGTCTCTGGAATATGTGTTCAAGGTGCAGGAAGTCCAGGAGAGGAAGATGTTTGACTTCGTAGAGCCGTTGCTGGCATTCCTTCAAGGGCTGTTTACTTACTACCATCATGGGTACGAGCTGGCCAAAGACTTCAACCACTTCAAGACAGACCTCACCATCAGCATTCAGAATACGCGGAACCGTTTTGAAAGCACACGGTCTGAGGTGGAGTGTCTGATGAGGAAGATGAAGGAGAACCCCCATGAGCACAAGAGTATCAGCCATGACACCATGGAGGGATACCTGTTTGTACAGGAGAAACGCTCTTTCGTGTCAACCTGGGTCAAGCACTACTGCACCTACCATCGAGAGCCCAAACGAGTCACCATGGTCCTGTTCGACCAGAAGTCGGGTGGCAAAGTGGGGGAGGAGGAGAGCTTCACTCTGAAGTCCTGCACAAGACGGAAAACGGATTCTATAGAGAAGAGGTTCTGTTTCGACGTGGAGGCTGTCGACAGGCCAGGGGTGATCACCATGCAAGCTCTATCGGAGGAAGATCGCAGGCTGTGGATGGAAGCTATGGATGGGAGAGAACCGGTCTATAACTTAAACAGAGACAGCCAGAGTGAAGGCCTGGCTCAGCTGGATGTGGTTGGCTTCAACGTTGTGAAGAAGTTCATCTACGCTGTGGAGACCAGAGGCATTGACGAGCAGGGCCTGTACAGGATTGTTGGAGTAAACTCCAGGGTGCAGAAACTCCTGGGTCTTGCCATGGATCCTAAAACCTGTGCTGACGTGGAGCTGGACAGTACAGAATGGGAGATCAAGACCATCACCAGCGCAATCAAGTACTACCTTAGAATGCTGCCCGCGCCCCTTATGACTTATCAGTATCAAAGGAGCTTCATCAAGGCAGCCAAACTGGACAATCAGGAGGCGAGGATCACAGAGATCCACAGCATCATCCACCGGCTCCCAGAGAAAAACCGCCAGATGCTGGACCTGCTTACCAAACACTTGGCAAATGTGGCCAGTCACCACCAGCAGAACCTGATGACTGTGGCCAACCTGGGCGTGGTGTTCGGCCCCACTCTGCTGAGGCCCCAGGAGGAGACTGTGGCTGCCATCATGGACATCAAGTTCCAGAACATTGTGGTAGAGATCCTCATCGAGAACCATGAGAGGATATTCAAGGATATGCCAGTTCCGGGAGGTGGCCAGGCCAACCTACAGCTCAACCTGCAGCCTCGGAGGAAAAGCGCAGAGAGCAAAGCCCCGTCCTGCAGCGAGAGACCCCTTACCCTCTTCCACACACCCACACACACCCAGAAAGTGGAGAAGAGGAACAGCGTGGTAGTGAACTCTACTCCAGAGCCCCAGCAGCTTCATCCGGCCCAGGCCAACGCCCCCCACAACAACCCATCAGCCACCCGCTCTTCCTCCACAGCAGCGGTCAACCATAACAGCAGCGGCAGCCACGGCCACAGCTGGACCCAACGCAACAGCCTGACCAGCACTGACGGAGATCAGGACGGGGGCCTGATGGCCAGGCAGAACTCACAGAGCCGCCCTAACTCACTGCTGAACCCAAAGAGCCACACCAGTATCCCAATGAGCCCAGCCTCACCGCGCTCCCCCTCCTGGCCGATGTTCTCTGCATCCTCCAGCCCCCAGCCGGCCTCCTCCACCTCCAGTGACTCCTCCCCCATCAGTGTCCCAGGCAGGAAGGCTAGGGCACTCTACGCCTGCAAGGCGGAGCACATCTCTGAGCTCTCGTTCATTGCAGGGACCATCTTTGAAAATGTTCATCACTCGAGGGAGCCTGGCTGGCTGGAAGGCATCCTAGACGGCAGGAAGGGCCTAATTCCAGAGAACTACGTGGAGTTTCTG

>comp124336_c1_seq9_CHORD1a

GCCAATATGGCACTTCTTTGCTACAACAAAGGCTGTGGAGAGAAGTTTGATGCCGACAAGAACAAAGACGATGCCTGTCTTCATCATCCAGGTGTTCCCATCTTCCACGATGCTCTGAAGGGTTGGTCTTGTTGTAGGAAGAGAACCACAGACTTCTCTGAATTCCTTTCTATCAAGGGCTGCACCCAGGGGCGTCATAGTAACGAAAAGCCCCAGGAGCCTATGCAGCCGACAGTGACATCAGACAAGAACGGTGTGAGAAACAACAGAGAGGAGATAATTTACCAGGGGCCCAAATCAGCTGAGGCACTGCAGAAAGAGAGACCCAGTTCAGATGAGGATAAGACCAAACTGAAGCTGAAGGTGTCCGCCTCTCTGACTCAGGTTCTGGAGAAGATGGAAATCAGCAGCAAGGCAGAACTAAAAAAGAAAGAGACTGCAGTTGTCATGCAGGGAACACGATGCAAGAACACAGGATGCAAAACACAATACCAGGGCCCAGAGACCAACGGAGAGACCTGTACACACCACCCTGGAGCACCTGTCTTCCACGAGGGGTATAAGTACTGGGGTTGCTGCTGTGTGAAGACCATAGACTTTAATGCTTTTCTGGATCAGAAGGGTTGTACCACTGGCAAACATCGCTGGGTCCAAAAAGTGAACAATAAGAAGGTTGCGTGTAGACATGACTGGCACCAGACAGCCACACAGGTCTACGTGACCATCTATGCCAAGAACGCCAACCCTGAACTGTCCTACGTCGAGGCTAACCGCACAGTGCTGACCTGTCACATCCAGTTTGAGGACAACAAGATTTTCCATAAGGACTACCATCTGTGGGGGGTAGCGGATGTAGCCAACAGTGGGGTCAACATGGTTTCGTCTAAGGTGGAAGTGTCGCTCCGTAAGGCAGATGCTGTGGCCTGGGGAAAACTAGAGGACCCCAAGTTCAAACCAGAACCCGAACCCCAGGATCTGGACATGGGTTACACCGATGAGGCCGACGACCCCAGTCAGTGGGACAGGGACATCGACGACGATGACATCAGTGACTCAGACGAGGAGTGGGAGAAACCGCAGATCATCCAGAATCCTCCAGAAGAGGATGAGATGCCCGTGTTAGAAGGC

>comp123038_c0_seq10_ckip1a

ATGAAGAAAAACAATTCAGCTAAACGGGGCCCGCAGGACACAAACCAGCACAACACGCAGCCAGACAAAATCGGTTGGATTCGCAAGTTCTGTGGCAAAGGGATATTTAGGGAGATCTGGAAAAATCGTTTTGTAATACTCAAAGGGGACCAACTTTACATCTCAGAGAAAGAGGTGAAGGATGAGAAGAAGATCCAGGAGGTGGTTGACCTGACGGATTACGAGCGTTCTGAGGAGCTCCGCAAGGCCAAGAGCCGCAGCAAGAAGAACCACAGCAAGTTCACCCTGCTGCGCTCCAGAACCCCAGGCAACACGGTCCCTAACCTGGTGTTCCTGGCTGTTAGTCCAGAGGAGAAGGAGTCCTGGATAAACGCCTTAAATGGAGCAATCACCAGATCCAAGAACAGCATCCTGGATGAGGTCATGGTTGAGGATGAAAACCTTCTGTCCCACCTCACACGGGACAGGGCTAAGATCCCTCACACACGGCGCCTGCCTACACGAGGACACATCATGGCTGTGGCCTCCACTTCTACCTCTGACGGCATGTTGACCCTTGACCTGATCCAGGAGGAGGACAGCCCCAGCACAGACGGCCAGGACACCTGTGACAAGAGCTTCCGGGCCAACCTGGACAAATCCACTCAGCTCGACTGCCTCAGGTCCATAACAGAGGGTGCCATCAGTAGCATCTGTCTAACGCCTCCCACAGAGGTCACTGGGAAGTCCCAGAGCCTGCCGCGCGATACCGGGGTCACCTGGGACGATAAACAGCAACTTCAGAGCCCGATGGGGAAGTCCTGCACTCCTCAGCCAGGCAAGAGGCTCACACCGGCCGAGAAAAGCCGCTGCGCCTCCATGGACGAGATCCTCTCACACTCCGGGACGCAGGCCACCAAACCCAGGACATCCATCCCCCGCTCTGCCACCTCTGCCCCCCCGGGCAACCTGCCGCCCATCAGCCAGCTGCAGGACCTAATCGCTCAGAAGCTGGAGCGAACTCAGGAGCTGCTGATGCAGGTCCAGGGGGCGAAGGGGGAGGAGGTGGAGCGGGGGAAGGGGAAAGGGAAGGACTCGCCAGGTTCCAAGGGCTCCAAGAGTTCCTCGGACGGGGCCCGGGCGGAGGCAGAGAGGCTGCTGAAGGAGGCAGCGTCCACATGGGGTCAGGCCAAGGATGTTCTAGAGGAGGTGAAGGAGCTGAGGGCGCTCTACCGTCAGCTGGACTCGCTGCCCACCACCCCTGCCTCCCTCTCACCCCTCACACCCTCTAACAGCGGCAAGCAGACTGACTACAGGAAGAGCATGATG

>comp132375_c2_seq13_trio

ATCAATGTAATCTCTCTCTCCCTTCCTCTTTCAGCCTCTGTCTCCCCCACTGTCTCTCTTTCTCACTCTCTCTCTCCAGCTCTGACCTCCCCTATAGAGTACCAGAGGATTCATGTAGGGGCTAGCGACGGACCATGTCTACCCAGCGGCTTAGCTCCGTCAGTGGGGAGCGTCATGCCCAGTGTGGCCCCTGTTGGAGGTCTACAGGGTGTGCCTCTCACTGGTCCCCTCGGTGTAGGTAGTCCCCTCATTGGAGGTGGTCCCCAGGGCGGAGCTGGCTCCTCCAGGAGGCCCTCCAGGATCCCTCAGCCCTCCCGCCTGCCCCAGCCCCTGCGACACCCACACCACCCGGGGGCCGCAGACCCAGACGGCCCCAACAAGATGTCAGGCTCCTCCCCTCGTCTCCCCCCTCATTCCTCGCTCCCTCACGCCGTCTCAGCAAACCCGGGGTCGCAGGCTCCAGCGGGGGGGGTGAGAGACACGAGGGAGGCCCAACGTGGGGTGAGGGTCCAGGGGGGCAGTCCCCAGGGCAAGCGCACATTCTCAGCCTCAACCGACCAGCACCCTCCCATCCCCCCTATTCCCCGAGCCAGTGTGGCCCCTCTCTCTGGGCCCGTGCTTCTTTCCACCCCCAGCAAGCCTCGCCCGGGCACCGTCTCCCCCATGGCCTCCCCCCTGGCAACTCCCGCCTTTGGGAAAGACGCCCTCCCGCCTCCCCCTCCCAGCCCTGGCCTGAAGTCTGGCTCCTTCTGGAGCTCCATGCCTGGTTCTCCAGCCAGTCGGCCCGGCTCCTTCACCTTCCCAGGGGAGACAGGAGAGACCATGGGGAGGCAGAACCAAAACACGTTCCAGACCTCCTCTCAGTCCGCGACAGGCTCCCACAGACACTCCACCCACAGCAAGGAGGCGGACCGCATGAGTACGTGCTCATCCACCAGCGAGCAGTCCACCCAGAGCAACGGGAGTGAAAGCAGCAGCAGCAGTAGTATGTCGACCATGTTGGTGACCCAGGACTACGTGGCCCTGAAGGAGGATGAGATCAGTGTTTCCCAGGGCGAGGTGGTCCAGATGCTGGCCTCCAATCAGCAGAACATGTTCCTGGTGTTCCGGGCTGCCACCGAGCAGGGCCCTGCCGCCGAGGGCTGGATCCCCGGCTACGTACTGGGTCACACCTCCACCATCATCCCCGACCTCCCCGAAGGAACCATCAAGAAGTCATCCTCGTGGCACACAGCACTCCGCATCAGGAGAAAGTCTGAGAGGAGAGATAAGGAGACCAGGAAGGAATATAAGATGGAAAACGGCTACCGCAAGTCTCAAGACTGTCTGACTAATAAAGTTTCTGTAAAGCTTCTAAACCCCAACTACATCTATGATGTACCCCCAGAGTTCCTGCTGCCTCTGAGTGATGTGACCTGTAACCTGGGGGAGAGTGTCACTCTGAGGAGTAAGGTCTGTGGAAGACCAAAAGCCTCGGTCACCTGGAGAGGATCTGACCACAGCACTCTGAGCAACGACGGACACTACAGCATCACCTACAGTGACACGGGTGAGGCAACTCTCCTCATCATGGGTGTGTCTGTGGAGGATGATGGGGTGTACACCTGTGTTGCCACCAACGTAGTGGGCAGCATGTCATCCTCCGCCAGCCTCAGAGTCTCAGAGGCCTCCGATGATGGGAGTGAAGTGATTTGGAAGAACAACTTTGAGTCCTTCTACACAGAGGTCACAGAACTGGGCAGGGGAAGGTTCTCAGTGGCTAAGCGCTGTGACCAGCGTGGGAGCAAGCGTGCCGTGGCAGCCAAACATGTCAACAAGCGGTTGATGCGGCGTGAGCAGGTGCTCCAGGAACTGAGGATACTACAGTGTCTGGAACACCCACACCTGGTCGGCCTGCTGGACACCTTCGAGACAGCCACCAGCTACGTGCTAGTGCTGGAGATGGCTGATCAAGGCCGTCTCCTGGACTACATAGTCAGCTGGGGTAACCTCACTGAAGAGAAGGTGGCCTTGTACCTTAGGGACATTCTAGAAGCTTTACACTACCTGCATAGCTGGAGGATAGCTCACTTGGACTTGAAGCCTGAGAACGTGTTAGTGGAGCAGAACTCTGCCCAGCCCGTGGTCAAGCTGACAGACTTCGGAGACGCCGTCCAGCTGAACAGTGGCCACTACATCCACCCCCTACTGGGCAGCCCAGAGTTCTCAGCCCCGGAGCTGGTGCTGGGCCAGCCCGTCTCCCTGACCTCTGACCTCTGGAGCCTGGGGGTTGTGACATACGTGGTACTGAGCGGGGCATCTCCCTTCCTGGACGAGAGCGTGGAGGAGACGTGTCTGAACATC

>comp126578_c0_seq1_vaspa

AAATCTGATGGGGGAGATGAGTGCCATCCTGGCCCGACGGAAGAAAATGTCAGATAATCCAGGCGCAAAGAAGGATGAGCCCGGCAATGAAGACTCCTCAAAGTCCTCAGGCCAAGGTGACACCCTCAGGAGACCGTGGGAGAAATCCGCCACCATGCCAAGGATGAAGACAGTGAACAGTACCACGGACAGCACAGGCAGTGAAGATACAGAGATGGAAAGAATCAAACAGGACATCCTTGAGGAAGTGAGAAAAGAGCTCCATAAAGTGAAGGATGAAATAATCTGTGCAT

>comp127832_c0_seq25_vaspb

CTTTCGTCTATCTTGTTCAGGAAAAAAATGGCAGACGGTGGGGCTAAGCCACCTGCTAAGACAGCAGATAATGATGACTCAGAGTCTCAAGGCCAAAGTGACACTCTTGGAAGAAGACCATGGGAGAAATCAGCCACTATGCCCAGGGTGAAGCATGCAGGTGGTAGTAATGATGCTGGAGGTGGTGAAGAGACAGATATGGAGAGGATTAAACAGGAGATTCTTGATGAGATGCGGAAGGAGTTACAAAAAGTCAAGGAGGAGATAATCGGAGCCTTTATTGAGGAGCTACAGAAGAGGGGTTCCACAT

>comp132062_c1_seq19_unc45b

ATGGGTGAGATGGCAGATCCAATCCAGTTCAAAGATGAGGGGAACAAGCATTTCCAAGCCGGTGAGGTCGACAAGGCCATTGAGTGTTACACTAAAGCCATCAAGTTGTGCACGGACAAAAAGGTTCTCGCCGTTGTCTACAGGAACAGATCAGCCTGTTTTCTGAAAAAGGAAAGCTACACCAATGCAGCCTCCGATGCCTCCAAAGCCATTGACGTGGATGCAGCAGACGTCAAAGCCCTGTTCCGGCGCTGCCAGGCTCTGGAGAAGCTGGGCAAGCTGGACATGGCCTTTAAAGATGTTCAGAGGTGTTCCACCATCGAGCCCAAAAACAAGACCTTCCTGGAGACCCTCAGGAGGCTGGGGGCTGAGATCCAAGCCAAGCTGAAGACAACATTCTCCACAGACTCACGGGTTCAGAACATGTTTGACATTCTGCTTGATGAGGAGATGGACAAGGACAAGAAGGAAATGGCTGCTAACAACCTGGTGGTTCTGGCCAGAGAAGAAGCTGGCGCAGAGAGAATCTTCCAGAACAATGGAGTGCCTCTGCTACTGGATTTGCTTGAGACCGGAAAAGTAGAGATGGTCCTGGCCGCTATCCGCACCTTCGCTGGAATGTGCACTGGACACAAAGCTCGGGCCATGGCCATTATCCACCTGGTGGGCATAGACAAGTTGTGCAGCATCATGGCCGTCGACAACGAGGACATTGCCTTGGCAACCTGTGACCTGTTCCAGTGCATCAATGACTCCCTCACTGGAGGAGACCGGAGGTTTTATGGGAAGGAGGAAGCCCTGGTTTTGGATGCAAGCAAGGACTTGAAGAGCATCCTGATGGCCCTGCTGGAGATGGTTGCCAGTAAGAAGGTTTCAGGACATGGCAGAGACCAGGCCCTGAACCTGCTGACCAGAAACGTGCCTCGCAAGGACAAGAAAGACCCTGACCACTCCAAGAGCCTCTTTACGATCGACCACGGTCTGAAGAAGATCCTGAAGGTGTGTGGCCAGATACCTGACCTCCCAGACCAGCTGCCCATGACAGAGAACACTCAGCTCATCGCCAGCGTGCTCCTCGACAAGCTCTGGGACGACCTGCGCTGCGACCCCGAGAGAGACAACTATAGGGAAGTCTGTGACGAGTACATCAAAGGCAAGTTTGACCCCAACGACATGGACAGGAACATCCACGCCATCAATGCTCTGTCAGGCCTGCTGCAGGGCCCCTTCGAGGTGGGCAACCAGCTGGTGGGTCGCCAGGGTATCATGGAGATGATGGTGGCGCTGTGCGGCTCCGAGCGTGAGGTGGACCAGATGGTCGCAGTGGAAGCACTGATCCACTCCTCGACCAAGATGAGCCGCGCCTCCTTCATCATCACCAACGGTGTGTCACTGCTCAAAGACATCTACAAGAAGACGAAGAACGATAAGATCAAAATCCGTGCGCTGGTGGGTCTGTGTAAGCTAGGCTCAGCAGGTGGAGATGACTATAGTATGAGGCAGTTTGCTGAAGGCTCCACTGAGAAACTGGCCAAGCAGTGTAGGAAATGGTTGTGTAACTCTGCCATGGACACGAGGACCAGGAAGTGGGCTATAGAAGGTTTGGCCTACTTAACCCAAGACGCTGATGTAAAAGATGACTTTGTTGAGGACGATCCTGCCATGAAAGCCATGTTTGACCTGGCCAAGTCTAAAGATAAGACCATCCTGTATGCTGTGGCCTGTACCCTGGTCAACTGCACCAACAGCTATGAGAAGAAAGACATCATGCCTGAGCTGGTTCAGCTGGCCAAGTTCTCCAAGCAGCACGTCCCTGAGCAGCACCCCAAGGACAAGAAAGATTTCATCCAGAAGAGAGTGAAGAGGATGCTAAAGGGAGGGGTCATCTCAGCTCTCACTGTCATGGTGAAAGCTGACAACGTTCTCCTGACTGACCAGACCAAAGAGATGCTGGCAAGGGTGTTCCTTGCCTTGGCAGATGATCCCAAAGACCGTGGCACTATTTGCGCCCATGGAGGGGGCAAGGCTCTGATCCCACTGGCTCTGGAGGGATCAGCTATAGGGAAAGTAAGGGCCGCACATGCCCTGGCCAAGATCGCTGCTGTCTCCAACCCGGAGATCGCATTCCCTGGAGAGAGGATCTATGAGGTGGTGCGTCCTCTGGTCAGCCTGTTGCACCCTGAAAGAGATGGGGTGCAGAACTACGAGGCACTTCTGAGTCTCACCAATTTGGCTGGTCTCAACGACAAACTGAGGGTGAAGATCCTGAAAGAGAAGGCTCTCCCTGAGATTGAGCAGTACATGTTTGAAGACCATGACCATATCAGACAGGCAGCCACAGAGTGCATGTGCAACCTGGTGACGTGCAAAGAGGTGCAGGATCGGTACCTGGAGGATGGGAATGATAGACTGAAGCTGCTGGTGCTGATGTGTGTTGAGGACAATGAAAAGCTCCAGAGAGCTGCAGCCGGGGGCCTGGCCATGCTCACCGCTGCCCAGAAGAAGCTGTGCACCAAGATGACCCTGGTGACGCTGCAGTGGATGGAGATCCTTCAGAGGCTGATTCTCCATGATCAGCCCCAGATTCAGCACAGAGGCCTTGTGATCGTGTACAACATGCTGAACTCGGACGACAACGAGCTGGCCAAGAAACTGATGGAGAGCGAGATCCTGGAGATCCTGACAGTGATTGGCAAAGCAATGGACAACCCCAAGAGGCAGATTGTAATCGATGTGGCACGCACCTGCCTGGTCAAGGCCATGGACCTCGGCCTCATCAAGCCCTTCACAACCCCT
